# Supplementary material for: Twenty-four-hour rhythmicities in disorders of consciousness are associated with a favourable outcome
Source: Commun Biol. 2023 Nov 29;6:1213. doi: 10.1038/s42003-023-05588-2 (PMC10687012; doi:10.1038/s42003-023-05588-2)
Supplement: Supplementary file 2 — Supplementary Information [file 42003_2023_5588_MOESM2_ESM.pdf]

Supplementary Figures

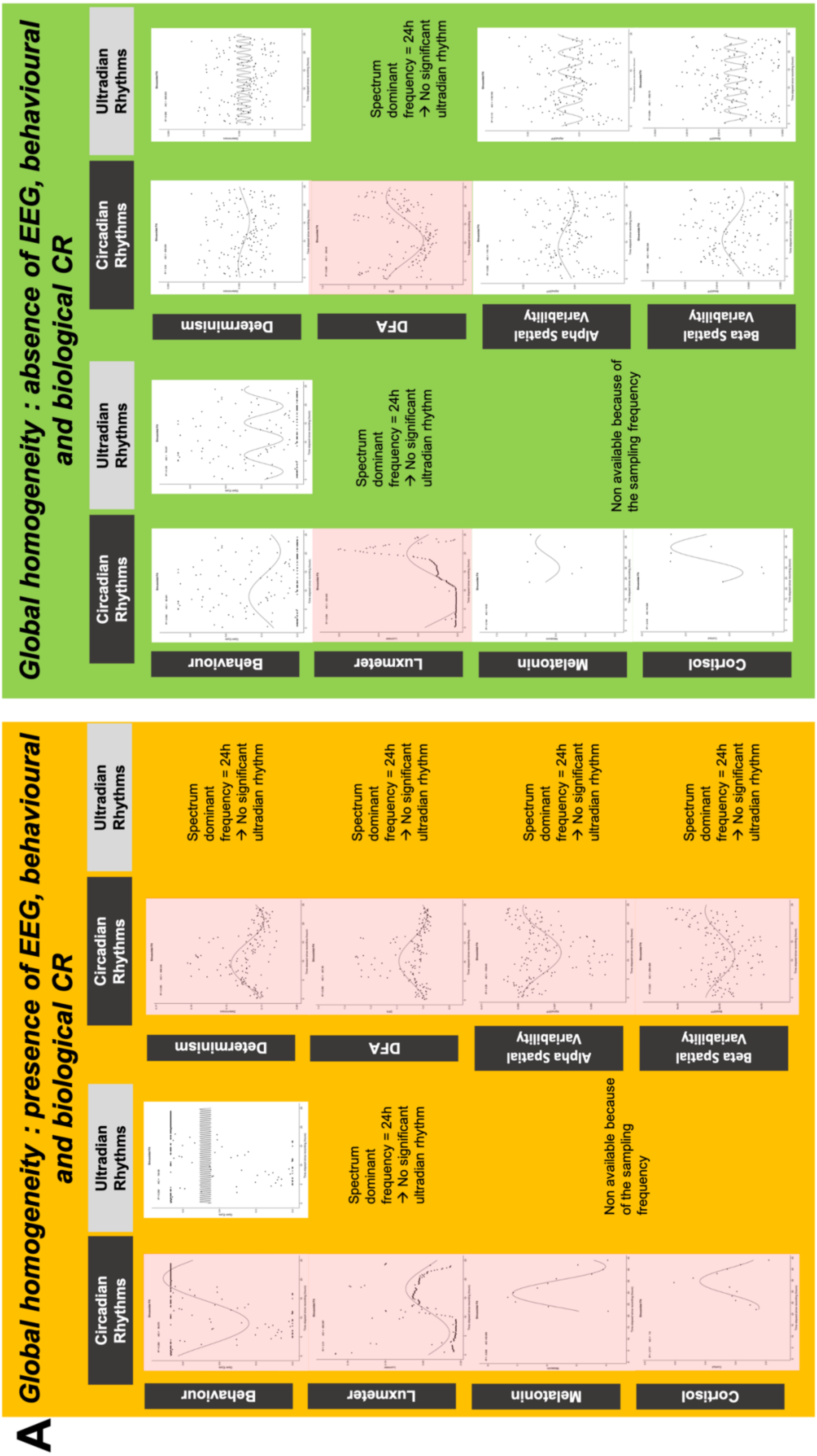

Supplementary Figure 1: ***Illustration of the four rhythmic patterns existing for healthy participants and patients during coma and post-coma***

Four possible situations are presented (see Fig. 5 for colour code):

A: “Homogeneous presence of all circadian rhythms” (orange) for Patient N°1, “Homogeneous absence of circadian rhythms” (green) illustrated by Patient N°12 (second recording) here and by Patient N°10 in Figure N°6-B.

B: “Emergence of abnormal EEG rhythms” (blue) for Patient N°14, and dissociation between behaviour and EEG (light blue) for Patient N°12 (first recording).

Only the scatter plots of raw data are presented with a common timescale in abscise (24h) and feature-specific ordinates for each line (with ad hoc scales for each plot). The name of each feature is indicated on the left (black box for the features presented for every patient; grey box for patient-specific EEG features). The four EEG features presented for every patient (Determinism, Detrended Fluctuation Analysis [DFA], Alpha Spatial Variability, Beta Spatial Variability) are those presenting a significant circadian rhythm for healthy participants. Hormonal plots present log transformed values. The fits for circadian (black box on the top) or ultradian (grey box on the top) rhythms are illustrated by sinusoidal curves (red frames for significant fits). The comparison with line curves presenting the fluctuations with a common scale is available at the individual level in Figure 6-A and Supplementary Figures 2-3-4.

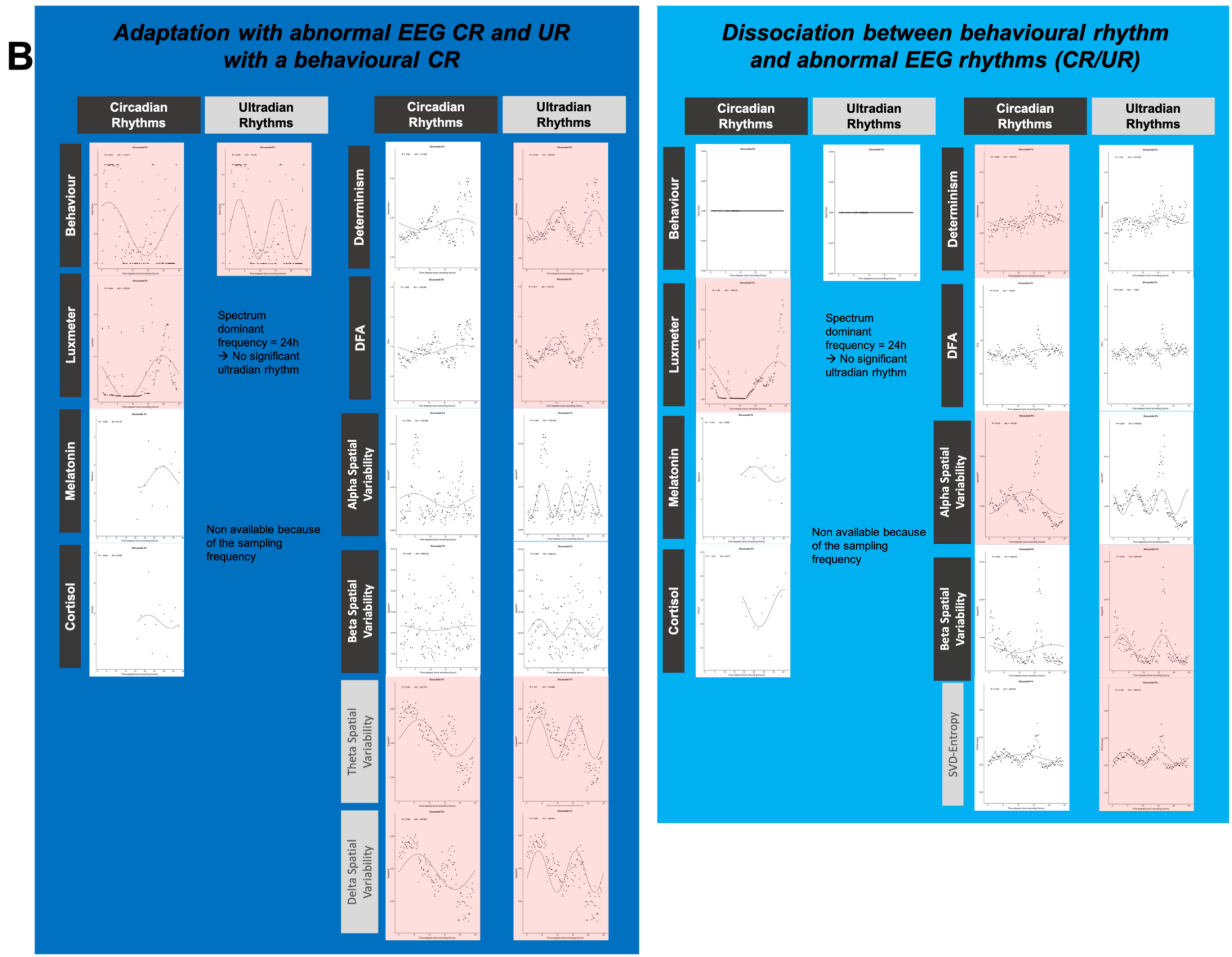
B
Adaptation with abnormal EEG CR and UR with a behavioural CR
Circadian Rhythms
Ultradian Rhythms
Behaviour
Luxmeter
Melatonin
Cortisol
Spectrum dominant frequency = 24h → No significant ultradian rhythm
Non available because of the sampling frequency
Determinism
DFA
Alpha Spatial Variability
Beta Spatial Variability
Theta Spatial Variability
Delta Spatial Variability
Dissociation between behavioural rhythm and abnormal EEG rhythms (CR/UR)
SVD-Entropy

<u>Supplementary Figure 2:</u> ***Illustration N°2 with Patient N°14 in Figure 5***

A singular case presenting a heterogeneity between the presence of behavioural circadian rhythm, the absence of any normal EEG circadian rhythm, but the existence of abnormal circadian rhythms (EEG) and ultradian rhythms (EEG and behaviour).

Patient 14 was a 71 y-o woman presenting a sub-arachnoid haemorrhage. GCS at admission was 8 without pupilar abnormalities. At the date of evaluation, the patient remained UWS/VS (with a CRS-R = 4 and GCS = 9). The neurophysiological battery was favourable. Considering the high risk of cognitive sequalae due to the existence of diffuse ischemic lesions related to a prolonged increased intra-cranial pressure (see Supplementary Table 2), awakening was assessed during 2 months then care was withdrawn in accordance with the family's wishes. She died at day 54 without having presented any behavioural sign of cortical function.

Raw data (continuous curves in the middle, with uniformed scales across each illustration), circadian fits (plots on the left, with ad hoc scales) or ultradian fits (plots on the right, with ad hoc scales) are presented for behavioural data (black), environmental data (yellow), hormonal data (blue for melatonin and orange for cortisol, with raw values for the continuous curves and log transformed values for the plots), and EEG with a common timescale in abscise (24h). The 4 EEG features (red: Determinism, blue: Detrended Fluctuation Analysis, violet: Alpha Spatial Variability, green: Beta Spatial Variability) are those presenting a significant circadian rhythm among healthy participants. EEG is defined as "circadian" when more than one feature presented a circadian fit. Plots with significant circadian and ultradian fits are indicated by red frames.

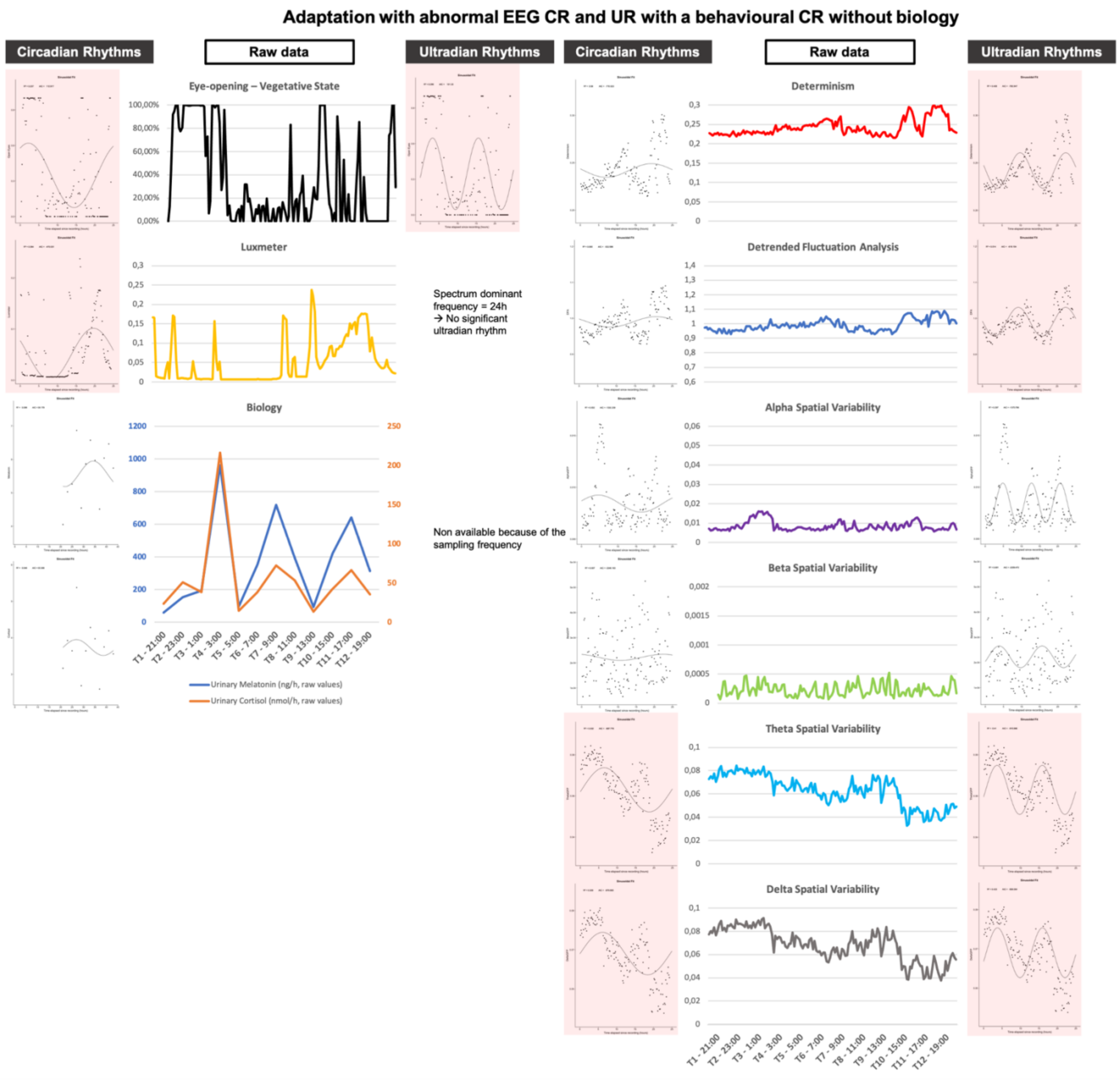

<u>Supplementary Figure 3:</u> *Illustration N°3 with Patient N°12 (first recording) in Figure 5*
A dissociated case presenting a complete absence of behavioural circadian rhythm and the existence of circadian rhythms for 2 among 4 EEG features observed in healthy participants, as well as abnormal ultradian rhythms for 2 EEG features.

Patient 12 was a 37 y-o man presenting a severe TBI. GCS at admission was 3 with a bilateral mydriasis. At the date of first evaluation, the patient remained comatose (with a CRS-R = 2 and GCS = 6). The neurophysiological battery was dubious, and the patient evolved eventually towards a MCS/CMS status after the second evaluation, in which he remained UWS/VS.

Raw data (continuous curves in the middle, with uniformed scales across each illustration), circadian fits (plots on the left, with ad hoc scales) or ultradian fits (plots on the right, with ad hoc scales) are presented for behavioural data (black), environmental data (yellow), hormonal data (blue for melatonin and orange for cortisol, with raw values for the continuous curves and log transformed values for the plots), and EEG with a common timescale in abscise (24h). The 4 EEG features (red: Determinism, blue: Detrended Fluctuation Analysis, violet: Alpha Spatial Variability, green: Beta Spatial Variability) are those presenting a significant circadian rhythm among healthy participants. EEG is defined as "circadian" when more than one feature presented a circadian fit. Plots with significant circadian and ultradian fits are indicated by red frames.

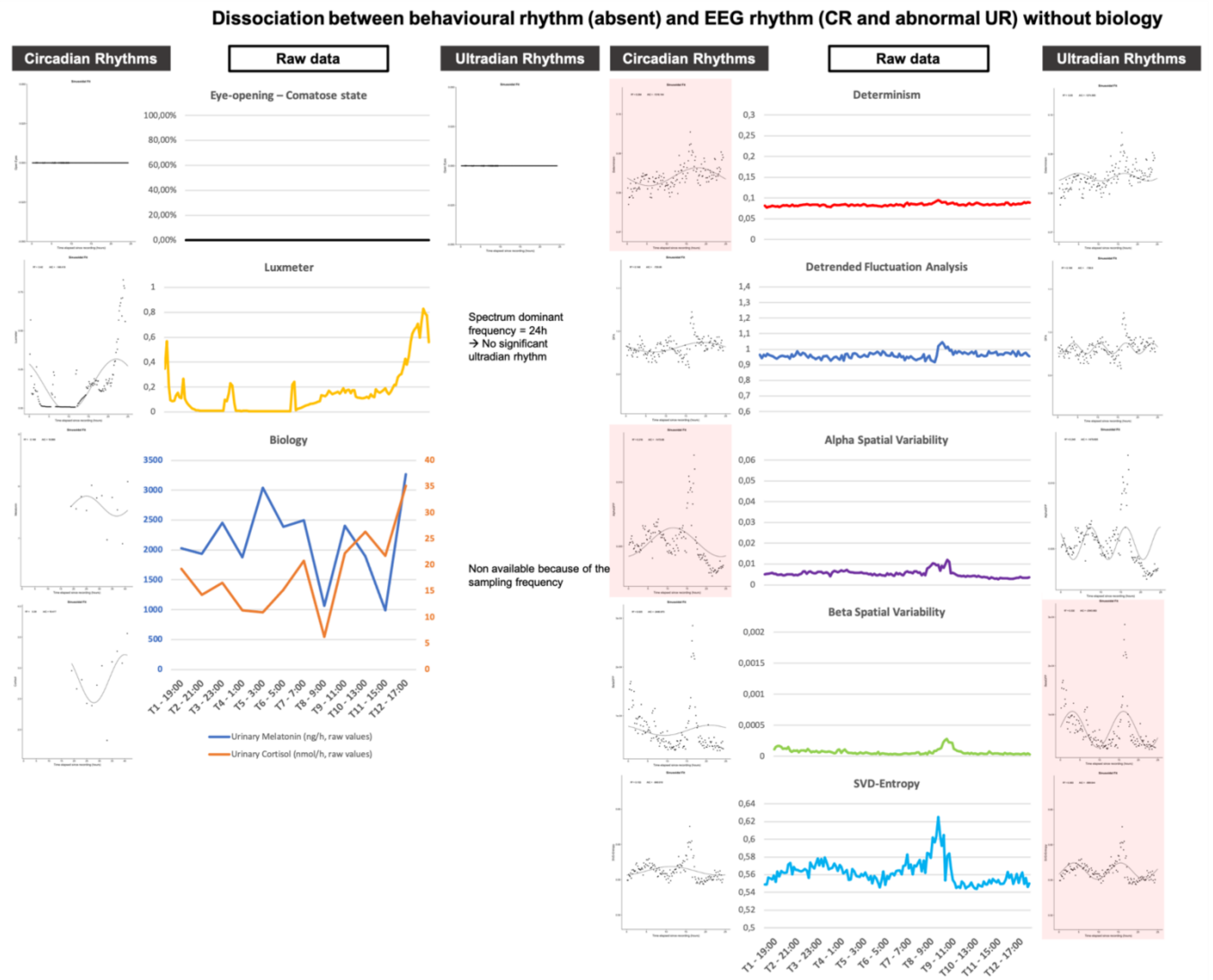

Supplementary Figure 4: ***Illustration of the normal rhythms assessed in a healthy participant***

Raw data (continuous curves in the middle, with uniformed scales across each illustration for EEG), circadian fits (plots on the left, with ad hoc scales) or ultradian fits (plots on the right, with ad hoc scales) are presented for behavioural data (black), environmental data (yellow) and EEG with a common timescale in abscise (24h). The 4 EEG features (red: Determinism, blue: Detrended Fluctuation Analysis, violet: Alpha Spatial Variability, green: Beta Spatial Variability) are those presenting a significant circadian rhythm among healthy participants. Plots with significant circadian and ultradian fits are indicated by red frames.

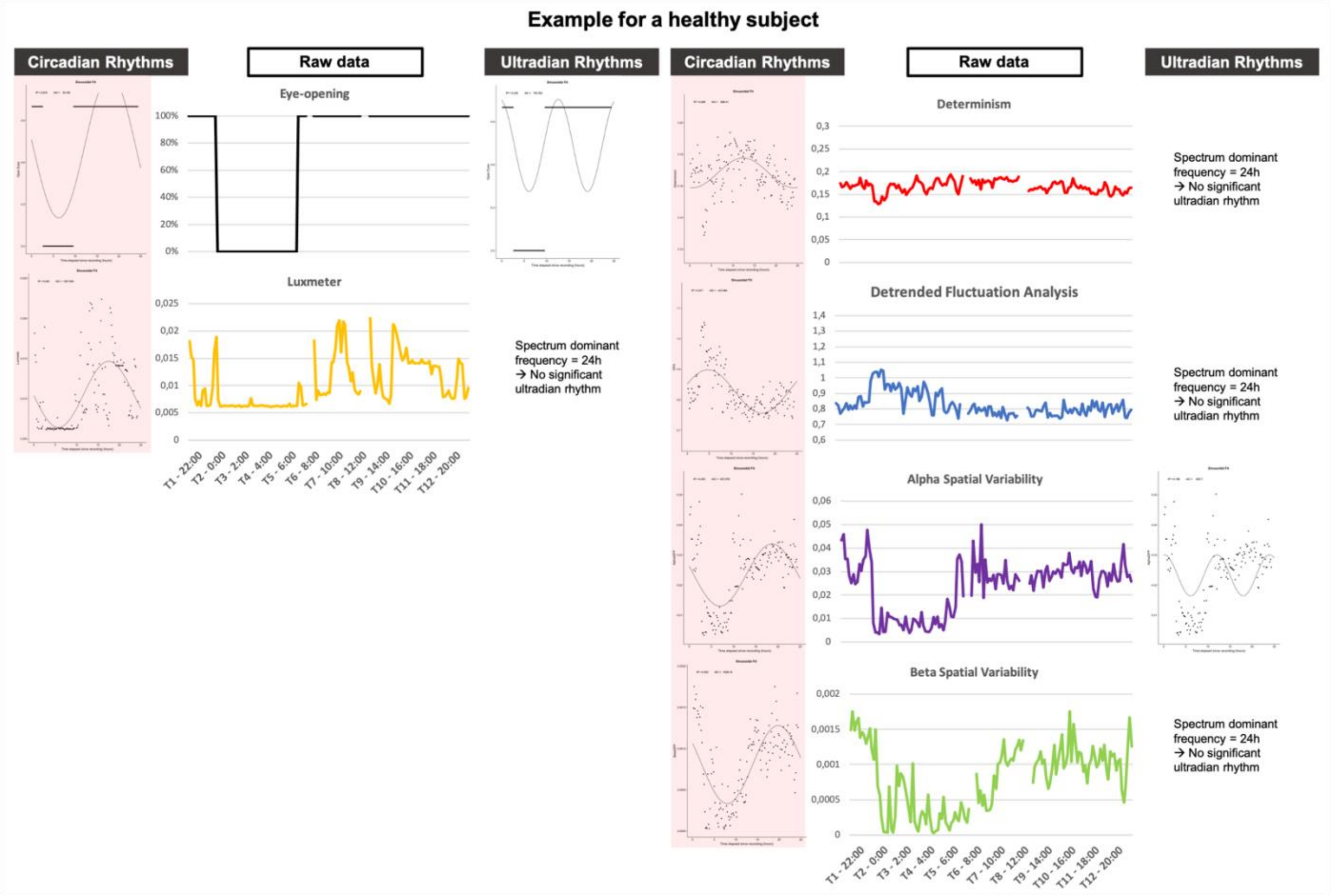

<u>Supplementary Figure 5:</u> ***Synthetic overview of homogeneity and heterogeneity between all circadian rhythms with details about abnormal circadian rhythms.***

Rhythm-based categorisation of patients (one patient per column, with the cause of coma on the top), using a combination of dichotomic assessments of circadian rhythms for hormones (yellow box), behaviour (orange box), and EEG (red box). Among each group, a qualitative description of abnormal circadian rhythms for EEG (red box), abnormal ultradian rhythms for EEG (red box), and abnormal ultradian rhythms for behaviour (orange box) is provided.
The colour code defining the group of homogeneous presence/absence of CR is the same for the examples given in Supplementary Figure 1.
Notably, the <u>"homogeneous presence of all circadian rhythms"</u> was associated with a favourable outcome among acute patients. The single exception concerned a chronic patient presenting an additional "abnormal ultradian EEG pattern" (Alpha Relative Power and SVD-entropy ultradian rhythms at a 6h15 period).
Patients' numbers are defined by their group of classification rather by the recording order.

CR: Circadian Rhythms
UR: Ultradian Rhythms
TBI: Traumatic Brain Injury
SAH: Sub-Arachnoid Haemorrhage
H: Hematoma
NA: Non-Available

Symbols for pathophysiological interpretations including the abnormal CR and UR patterns:
**: Homogeneity by presence of CR*
***: Homogeneity by absence of CR*
#: Dissociation between EEG and behaviour
##: Dissociation of biology
@: Adaptation with abnormal UR-CR

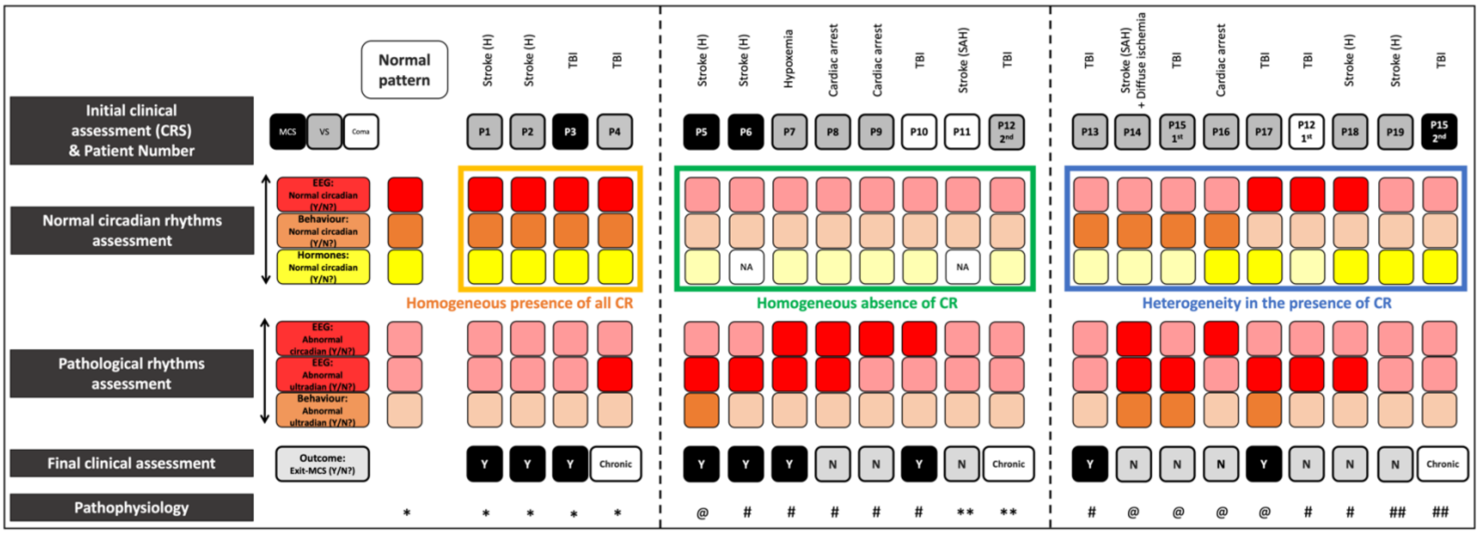

<u>Supplementary Figure 6:</u> ***Supervised data-driven analysis concerning the parameters related to the existence of a "Disorders Of Consciousness"***

Multidimensional classification of patients based on EEG parameters to perform a dichotomic segregation for the clinical factor **"Disorders Of Consciousness"**. Compared to healthy participants, DOC patients had a significantly *lower Dominant Period* for Determinism, a significantly *lower Coefficient of Variation* for Theta Spatial Variability, and a significantly *lower Standard Deviation* for Delta Spatial Variability.

Among the parameters univariately associated with the factor using a binomial logistic regression, the models with the 3 most accurate parameters were selected by a backward method minimising the AIC parameter. The relative distance between clusters is illustrated on the right (groups illustrated by coloured ellipsoids; patients illustrated by coloured dots of different sizes as a 3D-effect) and details for each parameter are illustrated by boxplots on the left. DOC patients are herein in red and healthy participants in grey.

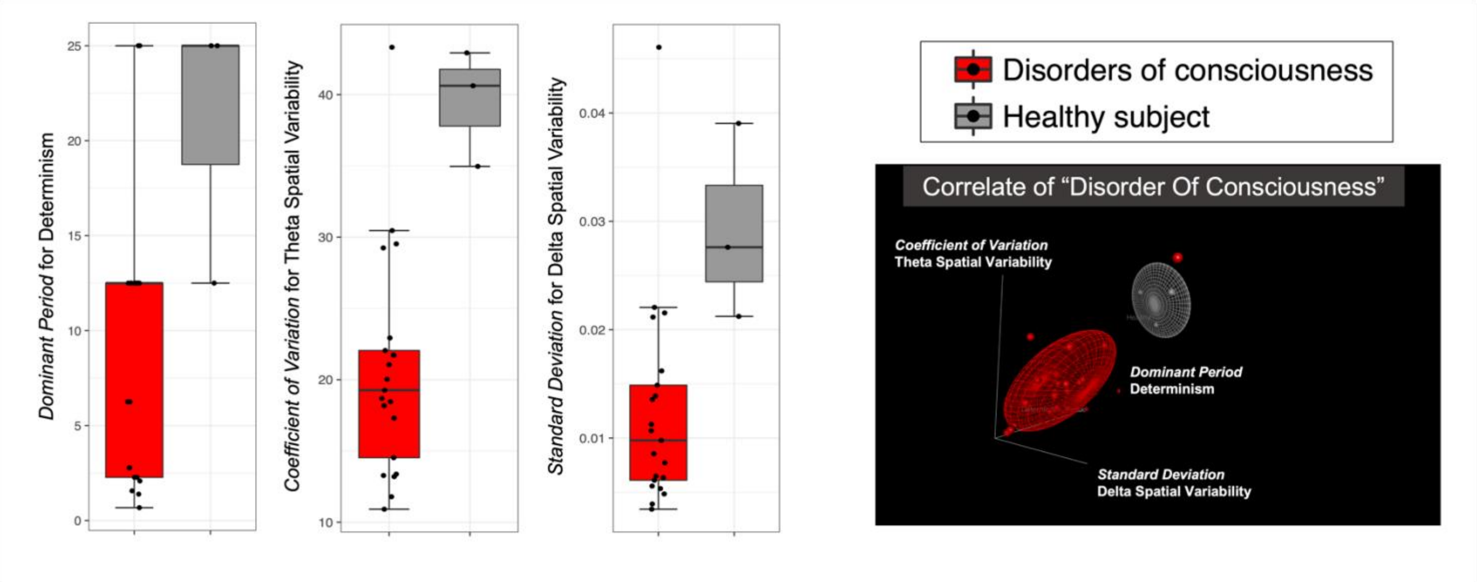

Factor	Disorders of consciousness	
Group	DOC patients	Healthy subjects
Parameter 1	Standard Deviation for Delta Spatial Variability (p = 0.0463)	
Values	0.012 (±0.0097)	**0.029 (±0.0091)**
Parameter 2	Coefficient of Variation for Theta Spatial Variability (p = 0.0394)	
Values	20.53% (±7.66)	**39.51% (±4.10)**
Parameter 3	Dominant Period for Determinism (p = 0.0452)	
Values	9.09h (±7.19)	**20.83h (±7.22)**

Supplementary Figure 7: ***Illustration of two EEG abnormal circadian rhythm patterns associated with an opposite outcome***

A strong circadian fit for the Alpha Dominant Frequency was associated with a favourable outcome for the 2 patients presenting this pattern after non-anoxic lesions (Patient N° 7 and 10). A strong circadian fit for the Alpha Relative Power was associated with an unfavourable outcome for the 3 patients presenting this pattern after a diffuse post-anoxic encephalopathy (Patient N° 8, 9, and 16).

The mechanisms of lesions are indicated on the left and illustrated by MRI sections of Diffusion Weighted Imaging for three patients with ischemic lesions and by Susceptibility Weighted Imaging for the single patient with post-traumatic diffuse axonal injuries (no MRI was available for one patient with a post-cardiac arrest renal failure with continuous renal replacement therapy).

Raw data (continuous curves in the middle, with common scales to compare the values across patients) and circadian fits (plots on the left side with ad hoc scales to emphasise the magnitude of fluctuations) are presented for Alpha Dominant Frequency (green) and Alpha Relative Power (red). Plots with significant circadian fits (R2 > 0.3) are indicated by red frames.

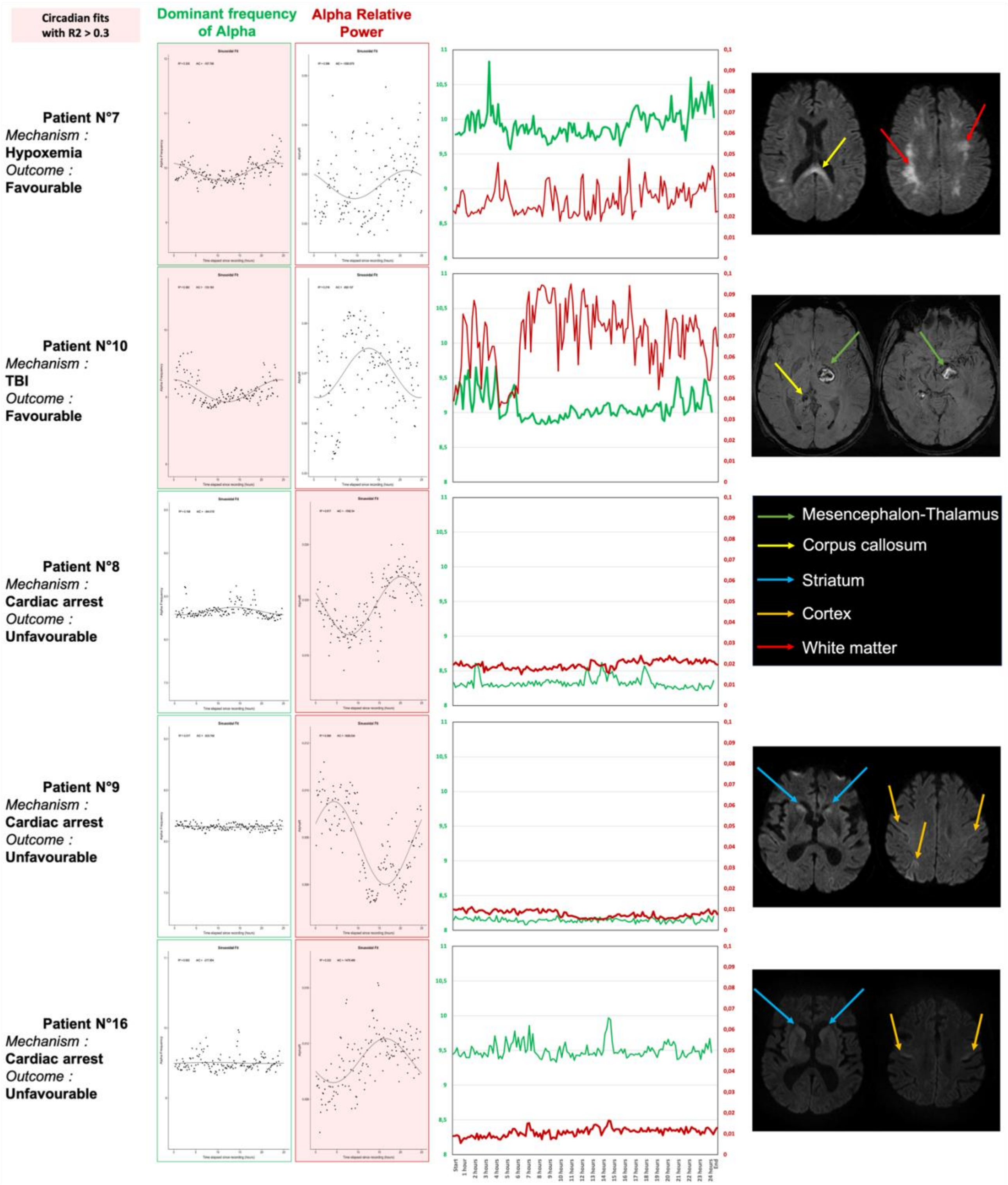

<u>Supplementary Figure 8:</u> ***Comparison of the best discriminative performances of quantitative clinical and neurophysiological variables for favourable outcome***

The 3 EEG parameters defined as the best predictors of outcome were systematically compared to a selection of multimodal variables: i) the clinical variables: age, initial status at admission (e.g. Glasgow Coma Scale), and at the date of 24h recording (e.g. Glasgow Coma Scale and Coma Recovery Scale-Revised; ii) the circadian fits for eye-opening periods; iii) the circadian fits of melatonin and cortisol urinary dosages.

After this selection (see Supplementary Table 6 for details), it appeared that the best associations of parameters included the *Permutation Entropy* for Beta Spatial Variability (common abscise). The following 2-dimentional comparisons have been selected for a visual illustration:
A: Comparison between the best EEG and clinical prognostic markers
B: Comparison among the best EEG prognostic markers
C: Comparison between the best EEG and hormonal prognostic markers

The patients presenting a favourable/unfavourable outcome are in blue and green, respectively. Chronic patients in red are illustrative (not taken into account for statistics). The orange dotted line separates linearly the clusters of patients defined by their outcome. The patient numbers came from the inclusion order and not the final classification used for interpretation in Fig. 5 and in Supplementary Figure 5.

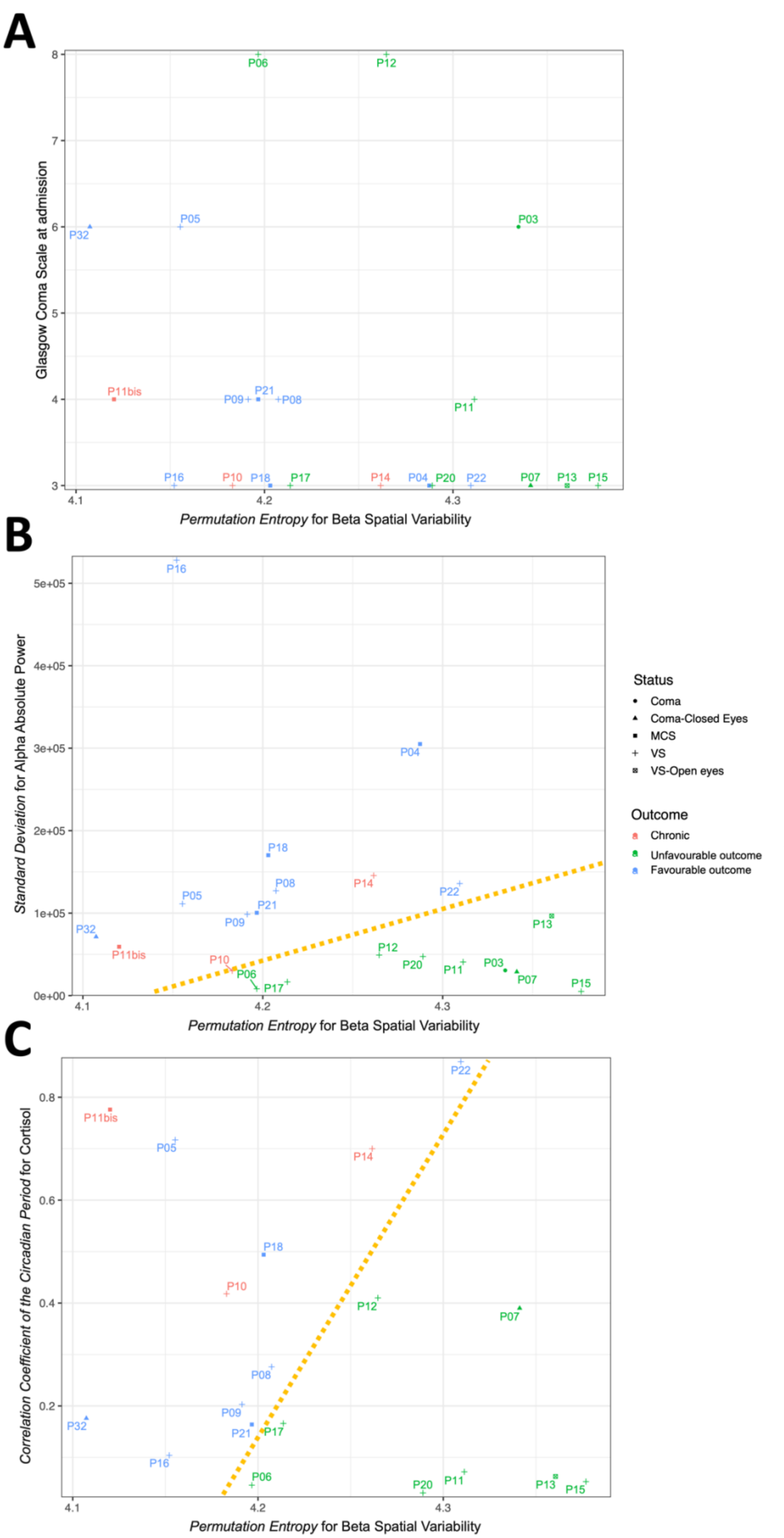

Supplementary Tables

Supplementary Table 1: ***Details of descriptive statistics***

CRS-R: Coma Recovery Scale – Revised
GCS: Glasgow Coma score
SD: Standard Deviation

	Mean	SD	Median	25th Percentile	75th Percentile	Minimum	Maximum
Age (years)	51	16.6	56	37	61	19	78
Delay after coma onset (days, for acute patients)	25.1	14.4	23	18	27	7	59
Delay after sedation withdrawal (days, for acute patients)	18.4	10	18	13	19	7	40
GCS at admission	4.2	2.1	4	3	5	3	8
GCS at the date of inclusion	8.4	1.5	9	7	9	5	10
CRS-R score at the date of inclusion	5.2	3.1	5	4	6	3	18

__Supplementary Table 2:__ ***Details of the population description for radiological features concerning cortical lesions (A) and extra-cortical lesions (B) with a synthetic view (C) for lateralised or bilateral lesions at the system level***

1: Lesions visible using T2 or Susceptibility-Weighted-Imaging only*
2: Lesions visible using FLAIR or T2SE only
3: Lesions visible using any imaging modality (T2 or Susceptibility-Weighted-Imaging) AND (FLAIR or T2SE)*
The groups of patients (illustrated by the respective colours) are defined according to Figure 5.
Stroke (H): Intra-parenchymal haematoma
Stroke (SAH): Sub Arachnoid haemorrhage

A

Patient Number	Age at admission (years)	Aetiology	Sex	Lateral Frontal		Medial Frontal		Central		Cingulate		Parietal		Temporal		Insular		Occipital		Corpus callosum	Corona Radiata		Internal Capsule	
				Right	*Left*	*Right*	*Left*	*Right*	*Left*	*Right*	*Left*	*Right*	*Left*	*Right*	*Left*	*Right*	*Left*	*Right*	*Left*	*na*	*Right*	*Left*	*Right*	*Left*
**P1**	58	Stroke (H)	M	0	0	0	3	0	3	0	3	0	3	0	0	0	0	0	3	0	0	0	0	0
**P2**	46	Stroke (H)	F	3	0	2	0	2	0	0	0	0	0	0	0	0	0	0	0	3	3	0	0	2
**P3**	36	TBI	M	0	3	0	0	0	0	0	0	0	2	0	2	0	0	0	2	3	0	0	0	0
**P4**	23	TBI	M	0	0	3	3	2	2	0	0	0	0	2	2	0	0	0	0	0	3	3	0	0
**P5**	56	Stroke (H)	M	0	3	0	0	0	0	0	0	0	3	0	3	0	0	0	0	2	0	3	0	3
**P6**	65	Stroke (H)	F	0	0	0	3	0	0	3	3	0	3	0	0	0	0	0	3	0	0	0	0	0
**P7**	30	Hypoxemia	M	2	2	0	0	0	0	0	0	2	2	2	2	0	0	0	0	2	2	2	0	0
**P8**	78	Cardiac arrest	M	na	na	na	na	na	na	na	na	na	na	na	na	na	na	na	na	na	na	na	na	na
**P9**	60	Cardiac arrest	M	0	0	0	0	0	0	0	0	0	0	0	0	0	0	0	0	0	0	0	0	0
**P10**	21	TBI	M	1	1	1	1	0	0	0	0	1	1	3	3	0	0	0	0	3	0	3	2	3
**P11**	60	Stroke (SAH)	F	0	3	2	3	0	0	0	0	0	0	0	0	0	2	0	0	3	0	0	0	0
**P12 (first)**<br>**P12 (second)**	37	TBI	M	3	3	0	0	0	0	0	0	0	0	1	3	0	0	0	0	3	0	3	0	0
**P13**	27	TBI	M	3	3	0	0	0	0	0	0	0	0	0	0	0	0	0	0	3	3	3	0	2
**P14**	71	Stroke (SAH)	F	2	2	2	2	2	2	2	2	2	2	2	2	0	0	2	2	2	0	0	0	0
**P15 (first)**<br>**P15 (second)**	38	TBI	M	3	3	0	0	0	0	3	3	0	0	0	3	0	0	0	0	3	0	1	2	2
**P16**	63	Cardiac arrest	F	0	0	0	0	0	0	0	0	0	0	0	0	0	0	0	0	0	0	0	0	0
**P17**	58	TBI	M	2	0	0	0	0	0	0	0	0	0	2	0	0	0	0	0	0	2	2	0	0
**P18**	49	Stroke (H)	M	0	0	0	0	0	0	0	0	0	0	0	0	0	0	0	0	0	0	0	0	0
**P19**	70	Stroke (H)	M	0	0	0	0	0	0	0	0	0	0	0	0	0	0	0	0	0	0	3	0	3

B

Patient Number	Age at admission (years)	Aetiology	Sex	Lateral Ventricle		3rd Ventricle	4th Ventricle	Caudate Nucleus		Putamen		Pallidum		Thalamus		Hypothalamus		Mesencephalon Tegmentum		Mesencephalon Tectum		Mesencephalon Pedonculus		Pons		Medulla oblongata		Cerebellum	
				Right	*Left*	*na*	*na*	*Right*	*Left*	*Right*	*Left*	*Right*	*Left*	*Right*	*Left*	*Right*	*Left*	*Right*	*Left*	*Right*	*Left*	*Right*	*Left*	*Right*	*Left*	*Right*	*Left*	*Right*	*Left*
**P1**	58	Stroke (H)	M	1	1	1	0	0	0	0	0	0	0	0	0	0	0	0	0	0	0	0	0	0	0	0	0	0	0
**P2**	46	Stroke (H)	F	0	1	0	0	2	0	2	0	0	0	2	2	0	0	0	0	0	0	0	0	0	0	0	0	0	0
**P3**	36	TBI	M	0	1	0	0	0	0	0	0	0	2	0	0	0	0	0	0	0	1	0	2	0	0	0	0	1	0
**P4**	23	TBI	M	0	0	0	0	0	0	0	0	0	0	2	3	0	0	2	2	2	2	2	2	0	0	0	0	0	0
**P5**	56	Stroke (H)	M	0	1	0	0	0	3	0	3	0	3	0	3	0	2	0	2	0	2	0	2	0	2	0	0	0	0
**P6**	65	Stroke (H)	F	0	1	1	0	0	0	0	0	0	0	0	0	0	0	0	0	0	0	0	2	0	0	0	0	0	0
**P7**	30	Hypoxemia	M	0	0	0	0	0	0	0	0	0	0	0	0	0	0	0	0	0	0	0	0	0	0	0	0	0	0
**P8**	78	Cardiac arrest	M	na	na	na	na	na	na	na	na	na	na	na	na	na	na	na	na	na	na	na	na	na	na	na	na	na	na
**P9**	60	Cardiac arrest	M	0	0	0	0	2	2	0	0	0	0	0	0	0	0	0	0	0	0	0	0	0	0	0	0	0	0
**P10**	21	TBI	M	1	1	0	0	0	0	0	3	0	3	0	3	0	3	2	2	0	3	2	3	0	3	0	0	0	3
**P11**	60	Stroke (SAH)	F	1	1	1	1	0	3	0	3	0	3	2	2	0	0	0	0	0	0	0	0	0	0	0	0	0	0
**P12 (first)**<br>**P12 (second)**	37	TBI	M	0	0	0	0	0	0	0	0	0	0	2	0	0	1	1	1	0	0	0	0	0	0	0	0	0	1
**P13**	27	TBI	M	0	0	0	0	0	0	0	3	0	2	0	0	0	0	0	0	0	0	0	0	0	0	0	0	0	0
**P14**	71	Stroke (SAH)	F	1	1	0	0	2	0	0	0	2	0	0	0	0	0	0	0	0	0	0	0	0	0	3	0	2	0
**P15 (first)**<br>**P15 (second)**	38	TBI	M	1	1	0	0	0	0	0	0	0	0	0	0	0	0	0	0	0	0	2	2	0	0	0	0	0	0
**P16**	63	Cardiac arrest	F	0	0	0	0	2	2	2	2	0	0	0	0	0	0	0	0	0	0	0	0	0	0	0	0	0	0
**P17**	58	TBI	M	0	0	0	0	0	0	0	0	0	0	0	0	0	0	0	0	0	0	0	0	0	0	0	0	0	0
**P18**	49	Stroke (H)	M	0	0	0	0	0	0	0	0	0	0	0	0	0	0	3	3	3	3	2	2	3	3	3	3	0	3
**P19**	70	Stroke (H)	M	1	1	1	1	0	3	0	3	0	3	0	3	0	3	0	0	0	0	2	0	0	0	0	0	0	0

C

Patient Number	Age at admission (years)	Aetiology	Sex	*Cortex / Hemispheric WM*	*Basal ganglia / Mesocircuit*	*Brainstem*	*Cerebellum*	*Corpus callosum*	*Hypothalamus*	*IVH*	*3rd Ventricle*	*4th Ventricle*
**P1**	58	Stroke (H)	M	Left	No	No	No	No	No	Bilateral	Yes	No
**P2**	46	Stroke (H)	F	Right	Right	No	No	Yes	No	Left	No	No
**P3**	36	TBI	M	Left	Left	Left	Right	Yes	No	Left	No	No
**P4**	23	TBI	M	Bilateral	Bilateral	Bilateral	No	No	No	No	No	No
**P5**	56	Stroke (H)	M	Left	Left	Left	No	Yes	Left	Left	No	No
**P6**	65	Stroke (H)	F	Left	No	Left	No	No	No	Left	Yes	No
**P7**	30	Hypoxemia	M	Bilateral	No	No	No	Yes	No	No	No	No
**P8**	78	Cardiac arrest	M	na	na	na	na	na	na	na	na	na
**P9**	60	Cardiac arrest	M	No	Bilateral	No	No	No	No	No	No	No
**P10**	21	TBI	M	Left	Left	Left	Left	Yes	Left	Bilateral	No	No
**P11**	60	Stroke (SAH)	F	Left	Left	No	No	Yes	No	Bilateral	Yes	Yes
**P12**	37	TBI	M	Left	Right	Bilateral	Left	Yes	Left	No	No	No
**P13**	27	TBI	M	Bilateral	Left	No	No	Yes	No	No	No	No
**P14**	71	Stroke (SAH)	F	Bilateral	Right	Right	Right	Yes	No	Bilateral	No	No
**P15**	38	TBI	M	Left	No	Bilateral	No	Yes	No	Bilateral	No	No
**P16**	63	Cardiac arrest	F	No	Bilateral	No	No	No	No	No	No	No
**P17**	58	TBI	M	Right	No	No	No	No	No	No	No	No
**P18**	49	Stroke (H)	M	No	No	Bilateral	Left	No	No	No	No	No
**P19**	70	Stroke (H)	M	Left	Left	Right	No	No	Left	Bilateral	Yes	Yes

<u>Supplementary Table 3:</u> ***Autocorrelations and originality between EEG features***

EEG features	Number of non-autocorrelation per subjects (mean)	EEG features	Number of non-autocorrelation per patients (mean)
Dominant Alpha Frequency	9.4	Dominant Alpha Frequency	9.00
Alpha Spatial Variability	8.1	Alpha Spatial Variability	7.95
Beta Spatial Variability	7.7	Beta Spatial Variability	7.33
Theta Spatial Variability	7.6	Theta Spatial Variability	6.86
Delta Spatial Variability	6.8	Delta Spatial Variability	6.57
AR4 Frequency	6.2	AR4 Frequency	6.38
Alpha Absolute	5.9	Alpha Absolute	6.00
Beta Absolute	5.9	Beta Absolute	5.81
AR4 Total	5.5	DFA	5.62
DFA	5.5	AR4 Total	5.19
Determinism	5.3	Determinism	5.19
Theta Absolute	5.1	Theta Relative	4.95
Theta Relative	5.0	Theta Absolute	4.90
SVD Entropy	5.0	SVD Entropy	4.86
Delta Relative	4.9	Delta Relative	4.67
Beta Relative	4.3	Beta Relative	4.29
Alpha Relative	4.3	AR4 Magnitude	4.00
AR4 Magnitude	4.0	Alpha Relative	3.95
Total Power	3.5	Total Power	3.48
Variance	3.3	Variance	3.33
Delta Absolute	3.3	Delta Absolute	3.29

A- At the feature-level: mean value of originality (no correlation) across subjects (patients and healthy subject on the left, patients only on the right). It is based on the number occurrences (per subjects or patients) for each feature of not being correlated with the others. A classification from the more original feature (top) to the less correlated feature (bottom) is provided. The features highlighted in yellow are the four ones which have been used to define a physiological EEG circadian rhythm, based on healthy subject.

Patient	Status	Outcome	Number of non-autocorrelated EEG features (mean)
P18	Coma	Non Favourable	10.1
P9	UWS/VS	Non Favourable	8.1
P17	UWS/VS	Favourable	8.1
P1	UWS/VS	Favourable	7.9
S01	Conscious	Healthy	7.0
P13	UWS/VS	Favourable	6.9
S02	Conscious	Healthy	6.8
P4	UWS/VS	Chronic	6.5
P14	UWS/VS	Non Favourable	6.3
P21	MCS/CMS	Chronic	6.0
S03	Conscious	Healthy	6.0
P7	UWS/VS	Favourable	5.8
P3	MCS/CMS	Favourable	5.8
P2	UWS/VS	Favourable	5.0
P10	Coma	Favourable	5.0
P16	UWS/VS	Non Favourable	4.8
P19	UWS/VS	Non Favourable	4.7
P8	UWS/VS	Non Favourable	4.7
P6	MCS/CMS	Favourable	4.6
P15	UWS/VS	Non Favourable	4.4
P11	Coma	Non Favourable	4.1
P12	UWS/VS	Chronic	4.1
P5	MCS/CMS	Favourable	1.0
P20	UWS/VS	Non Favourable	0.0

B- At the subject-level: mean value of originality (no correlation) across features. It is based on the number of occurrences (per patients) of not having correlated EEG features. A classification from more occurrence (top) to less occurrence of original features (bottom) is provided. Values in blue are above (mean + 1.96 SD of healthy subjects). Values in red are below (mean - 1.96 SD of healthy subjects).

C- Description of the number of occurrences for original (not correlated) EEG features among patients' recordings (healthy subjects excluded) presented per group of features (see Figure 1).

	Power		Spectrum												Complexity			Spatial Variability			
	Total Power	Variance	Beta Absolute	Alpha Absolute	Theta Absolute	Delta Absolute	Beta Relative	Alpha Relative	Theta Relative	Delta Relative	Dominant Alpha Frequency	AR4 Magnaitude	AR4 Frequency	AR4 Total	Determinism	DFA	SVD Entropy	Beta Spatial Variability	Alpha Spatial Variability	Theta Spatial Variability	Delta Spatial Variability
Total Power	na	0	4	1	3	0	0	1	2	0	10	1	6	4	2	5	5	5	11	7	6
Variance		na	4	2	3	0	0	1	3	1	9	0	6	2	2	4	3	7	10	6	7
Beta Absolute			na	2	4	3	9	8	8	9	9	6	7	6	3	4	7	8	8	6	7
Alpha Absolute				na	1	2	7	8	8	9	9	9	12	7	0	4	7	10	10	10	8
Theta Absolute					na	3	5	4	6	6	8	4	8	6	0	4	7	7	11	7	6
Delta Absolute						na	0	0	1	0	9	0	5	4	2	5	4	6	10	7	8
Beta Relative							na	0	1	2	9	1	6	3	3	7	3	8	8	9	9
Alpha Relative								na	1	0	9	0	2	4	6	8	1	9	7	7	7
Theta Relative									na	0	10	6	2	8	6	5	0	10	9	8	10
Delta Relative										na	10	4	2	8	7	5	0	10	9	7	9
Dominant Alpha Frequency											na	9	11	8	9	10	8	10	13	10	9
AR4 Magnitude												na	6	0	5	8	3	5	6	5	6
AR4 Frequency													na	9	8	5	3	9	8	10	9
AR4 Total														na	4	7	5	6	8	6	4
Determinism															na	3	10	10	13	8	8
DFA																na	6	6	8	7	7
SVD Entropy																	na	9	5	8	8
Beta Spatial Variability																		na	3	9	7
Alpha Spatial Variability																			na	7	3
Theta Spatial Variability																				na	0
Delta Spatial Variability																					na

Unit: number of occurrences for original (not correlated) EEG features
The maximum value in each case is 21 (number of recordings).

D- Synthetic view about the originality of the comparison between groups of EEG features.

Inter-Group	Power-Spectrum	Power-Complexity	Power-Spatial Variability	Spectrum-Complexity	Spectrum-Spatial Variability	Complexity-Spatial Variability
Mean	2.63	3.50	7.38	4.81	8.10	8.08
Rank	9	8	3	4	1	2
**Intra-Group**	**Power**	**Spectum**	**Complexity**	**Spatial Variability**		
Mean	0.00	4.76	4.22	3.63		
Rank	10.00	5	6	7		

Unit: number of occurrences for original (not correlated) EEG features
The maximum value in each case is 21 (number of recordings).

Supplementary Table 4: ***Environmental assessments***

The circadian status (presence or absence) is provided for sonometer (left) and luxmeter (middle) data. The Spearman correlations between behavioural and environmental assessments (Rho coefficients and p-values) are provided for sonometer (left), luxmeter (middle), and nursing (right) data.

Patients	Correlation with Eye-opening / **Sonometer**			Correlation with Eye-opening / **Luxmeter**			Correlation with Eye-opening / **Nursing**	
	Presence of a CR	Rho-values	p-values	Presence of a CR	Rho-values	p-values	Rho-values	p-values
P1	Yes	0.45	**1.51E-08**	Yes	0.26	1.35E-03	0.09	2.85E-01
P2	Yes	0.35	**1.76E-05**	Yes	0.50	**1.02E-10**	0.39	**1.14E-06**
P3	Yes	0.01	9.48E-01	Yes	0.44	**3.79E-08**	0.40	**6.93E-07**
P4	Yes	0.29	5.13E-04	Yes	0.54	**1.45E-12**	0.32	**1.04E-04**
P5	Yes	0.24	3.84E-03	Yes	0.00	9.82E-01	0.22	1.02E-02
P6	Yes	0.01	9.43E-01	Yes	0.16	4.88E-02	0.64	**2.20E-16**
P7	No	-0.11	1.74E-01	Yes	-0.10	2.41E-01	-0.23	5.35E-03
P8	No	0.23	5.10E-03	Yes	0.12	1.43E-01	0.24	2.81E-03
P9	No	0.38	**1.63E-06**	Yes	0.20	1.59E-02	0.32	**8.18E-05**
P10	Yes	NA	NA	Yes	NA	NA	NA	NA
P11	Yes	0.19	2.13E-02	Yes	0.26	1.64E-03	0.48	**3.23E-10**
P12 (bis)	Yes	-0.14	8.36E-02	Yes	-0.27	9.50E-04	-0.13	1.20E-01
P13	Yes	0.20	1.59E-02	Yes	0.35	**1.34E-05**	0.13	1.22E-01
P14	No	0.00	9.95E-01	Yes	-0.01	9.18E-01	0.17	3.89E-02
P15 (first)	Yes	-0.20	1.71E-02	Yes	-0.29	3.20E-04	0.02	7.76E-01
P16	No	0.17	3.55E-02	Yes	0.10	2.08E-01	0.18	3.07E-02
P17	Yes	0.27	9.73E-04	Yes	-0.25	1.84E-03	0.27	9.04E-04
P12 (first)	Yes	NA	NA	Yes	NA	NA	NA	NA
P18	Yes	-0.13	1.03E-01	Yes	-0.13	1.06E-01	-0.49	**1.42E-10**
P19	No	0.09	2.90E-01	Yes	0.32	**4.69E-05**	0.23	3.99E-03
P15 (bis)	Yes	0.12	1.49E-01	Yes	0.11	1.73E-01	0.36	**7.25E-06**
Healthy subjects								
S01	Yes	0.611	2.20E-16	Yes	0.762	2.20E-16	NA	NA
S02	Yes	0.741	1.44E-15	Yes	0.717	2.20E-16	NA	NA
S03	Yes	0.126	2.20E-16	Yes	0.122	2.20E-16	NA	NA

The patients are presented per group according to the classification proposed in Figure 5 ("Homogeneous presence of all circadian rhythms" in orange, "Homogeneous absence of circadian rhythms" in green and "Heterogeneity in the presence of circadian rhythms" in blue).
Significant p-values after Bonferroni correction are in red.

CR: Circadian Rhythms
NA: Non available

<u>Supplementary Table 5:</u> ***Comparison of the predictive values for clinical and neurophysiological qualitative markers (dichotomic)***

Two complementary levels of outcome were considered:
- On the left: **functional outcome** segregating the final outcome after a 2-year follow-up as “Favourable” for patients at least Exit-MCS (including severe disability, moderate disability, and good recovery) and “Unfavourable” for patients at best MCS (including coma, UWS/VS, and MCS/CMS).
- On the right: **awakening outcome** segregating the last available outcome after a 2-year follow-up as “Behavioural Cortical Function” for patients at least MCS (including MCS/CMS, severe disability, moderate disability, and good recovery) and “No Behavioural Cortical Function” for patients at best UWS/VS (including coma and UWS/VS).

The **clinical markers** tested were: “Behavioural cortical function”, “Mechanism of lesions”, “Existence of behavioural Circadian Rhythms”, and “Existence of biological Circadian Rhythms”.
The **neurophysiological markers** tested were: “Normal SEPs morphology”, “Normal BAEPs morphology”, “Normal MLAEPs morphology”, “Bilateral and Multimodal abolition of primary cortex response”, “Existence of N100”, “Existence of Mismatch Negativity”, “Existence of P300 response”, “Existence of MMN or P300 response”, “Existence of EEG reactivity”, “Existence of normal EEG Circadian Rhythms”, “Existence of abnormal EEG Circadian Rhythms”, and “Existence of abnormal EEG Ultradian Rhythms”.
The **composite markers** tested were: “Homogeneous presence of all circadian rhythms”, “Combination between EEG reactivity OR Homogeneous presence of all circadian rhythms”, and “Combination between Initial Clinical Status OR Homogeneous presence of all circadian rhythms”.

	Functional outcome (≥ Exit-MCS)		Statistics (selection of variable with X-squared p-values < 0.1)
	Favourable	Unfavourable	
Behavioural cortical function (CRS)	Se = 33.3%	Sp = 100%	Fisher's Exact Test = 0.206
Presence	3	0	PPV = 100%
Absence	6	9	NPV = 60%
EEG Reactivity	Se = 100%	Sp = 44.4%	Fisher's Exact Test = 0.082
Presence	9	5	PPV = 64.3%
Absence	0	4	NPV = 100%
Homogeneous presence of all circadian rhythms	Se = 37.5%	Sp = 100%	Fisher's Exact Test = 0.2
Presence	3	0	PPV = 100%
Absence	5	8	NPV = 61.5%
EEG Reactivity OR Homogeneous presence of all circadian rhythms	Se = 100%	Sp = 44.4%	Fisher's Exact Test = 0.082
At least one present	9	5	PPV = 64.29%
Both absent	0	4	NPV = 100%
**Behavioural cortical function OR Homogeneous presence of all circadian rhythms**	**Se = 55.6%**	**Sp = 100%**	**Fisher's Exact Test = 0.029**
At least one present	**5**	**0**	**PPV = 100%**
Both absent	**4**	**9**	**NPV = 69.2%**

	Awakening outcome (≥ MCS)		Statistics (selection of variable with X-squared p-values < 0.1)
	Cortical function	No cortical function	
EEG Reactivity	Se = 91.7%	Sp = 50%	Fisher's Exact Test = 0.083
Presence	11	3	PPV = 78.6%
Absence	1	3	NPV = 75%
EEG Reactivity OR Homogeneous presence of all circadian rhythms	Se = 91.7%	Sp = 50%	Fisher's Exact Test = 0.083
At least one present	11	3	PPV = 78.6%
Both absent	1	3	NPV = 75%
Behavioural cortical function OR Homogeneous presence of all circadian rhythms	Se = 41.7%	Sp = 100%	Fisher's Exact Test = 0.114
At least one present	5	0	PPV = 100%
Both absent	7	6	NPV = 46.2%
**Mechanism of lesions**	**Se = 91.7%**	**Sp = 66.7%**	**Fisher's Exact Test = 0.022**
Multifocal lesions (stroke, TBI)	**11**	**2**	**PPV = 84.6%**
Diffuse lesions	**1**	**4**	**NPV = 80%**
Primary cortical response	Se = 100%	Sp = 33.3%	Fisher's Exact Test = 0.098
No bilateral multimodal abolition	12	4	PPV = 75%
Bilateral multimodal abolition	0	2	NPV = 100%
Abnormal EEG Circadian Rhythm	Se = 83.3%	Sp = 66.7%	Fisher's Exact Test = 0.107
Absence	10	2	PPV = 83.3%
Presence	2	4	NPV = 66.7%
Morphology of MLAEPs	Se = 75%	Sp = 66.7%	Fisher's Exact Test = 0.141
Normal	9	2	PPV = 81.8%
Any reduction of amplitude	3	4	NPV = 57.1%

Chi2 tests were used as a non-parametric statistic to identify the markers strongly associated with the outcome in both categories. Only the markers with a trend (p-value < 0.1) are presented on both sides. The Fisher’s Exact test was used as a more stringent criteria due to the reduced sample of 18 acute patients (p-value < 0.05; significant prognostic markers are in bold red).

CRS-R: Coma Recovery Scale – Revised
EEG: Electroencephalogramm
BAEPs: Brainstem Auditory Evoked Potentials primary cortices
SEPs: Somatosensory Evoked Potentials
MLAEPs: Middle Latency Auditory Evoked Potentials
N100: Negative response at 100 ms for oddball auditory stimulus
MMN: Mismatch Negativity
P300: Positive response at 300 ms for subject own name stimulus
MCS: Minimally Conscious State
Se: Sensitivity
Sp: Specificity
PPV: Positive Predictive Value
NPV: Negative Predictive Value

<u>Supplementary Table 6:</u> ***Comparison between clinical and multimodal quantitative markers***

A selection of 3 EEG parameters was associated with circadian behavioural and hormonal markers to predict the favourable outcome. They were compared to other outcome predictors (with MANOVA p-values indicated in each corresponding cell). On the bottom, the comparisons between every marker from clinical or non-clinical groups illustrate that an EEG-based categorisation was more effective.

EEG/Clinical markers	Age	Initial GCS	Initial pupil	GCS at recording	CRS-R at recording
***Standard Deviation* for Alpha Absolute Power**	0.0143	0.0369	0.0339	0.0245	0.006
***Standard Deviation* for Alpha Spatial Variability**	0.0774	0.1621	0.1137	0.097	0.0245
***Permutation Entropy* for Beta Spatial Variability**	0.0091	**0.0027**	0.0139	0.0077	0.0049

Non Clinical markers	Behaviour CR	Melatonin CR	Cortisol CR	**AlphaA Std**	**AlphaR Variability Std**	**BetaR Variability PE**
Correlation Coefficient of the Circadian Period for Behaviour						
Correlation Coefficient of the Circadian Period for Melatonin	0.9237					
Correlation Coefficient of the Circadian Period for Cortisol	0.2006	0.2058				
***Standard Deviation* for Alpha Absolute Power**	0.0181	0.0973	0.0151			
***Standard Deviation* for Alpha Spatial Variability**	0.1138	0.1823	0.024	0.0315		
***Permutation Entropy* for Beta Spatial Variability**	0.0216	0.0224	**0.0023**	**0.0019**	0.0091	

Clinical markers	Age	Initial GCS	Initial pupil	GCS at recording	**CRS-R at recording**
Age					
Initial Glasgow Coma Score	0.1818				
Initial pupil status	0.1794	0.7205			
GCS at recording	0.1358	0.7574	0.7969		
**CRS-R at recording**	0.0406	0.1239	0.1174	0.1161	

Clinical & Non Clinical markers	0.0331
All Clinical markers	0.192
All Non Clinical markers	0.0423
All Non EEG markers	0.3733
All EEG markers	**0.0042**

The type of comparison is highlighted in yellow in each part of the table. The association of markers associated with the outcome in univariate analysis are in bold. The Bonferroni-corrected p-values (MANOVA test for comparisons between-markers) are in bold red.

CRS-R: Coma Recovery Scale – Revised
GCS: Glasgow Coma score
EEG: Electroencephalogramm
PE: Permutation Entropy
A: Absolute
R: Relative
Std: Standard deviation
CR: Circadian Rhythms

Supplementary Text

Supplementary data 1: Radiological features of patients' lesions

All patients but one (with a contra-indication to MRI during the ICU course) underwent an MRI-based evaluation of the lesion responsible for the delayed awakening in the weeks before or after his/her inclusion in the present study. This description of lesions is provided at the individual level in Supplementary Table 2 for cortical and hemispheric white matter lesions (Part A) and for the sub-cortical areas and the ventricular system (Part B).

The synthetic view provided in part C allowed a qualitative description according to the groups of patients defined in the main text and in Fig. 5. The group of 4 patients presenting a "homogeneous presence of all CR" was only specific by the absence of any visible lesion in hypothalamic areas (at the resolution provided by clinical MRI that were not focused on this region).

At the population-level, the occurrence of median (corpus callosum) involvement were the most common (52.6%), followed by left predominance of lesions (27.4%), bilateral lesions (11.6%) and right predominance of lesions (9.5%).

For the "homogeneous presence of all CR" group, the results were slightly below those for the entire population for left (20%) and median (50%) lesions and above for right (15%) and bilateral (15%) lesions.

For the "homogeneous absence of all CR" group, the results were slightly above those for the entire population for left (40%) and median (62.5%) lesions and below for right (2.5%) and bilateral (7.5%) lesions.

For the "heterogeneity in the presence of CR" group, the results were slightly below those for the entire population for left (17.1%) and median (42.9%) lesions and above for right (14.3%) and bilateral (14.3%) lesions.

Supplementary data 2: Autocorrelations between EEG times series to describe the originality of features

As excepted because EEG features give only a different (but possibly complementary) view of the same physical process (a synthetic electo-ionic function of the brain), autocorrelations across features may occur.

We performed a dedicated analysis in the initial steps of EEG processing/analysis, to address the question of the “originality” of each EEG features, according to the correlations between time series (two-by-two, Spearman correlations, adjusted P-values based on Holm’s method). The originality of one particular EEG features compared to the other was defined as the “absence of correlation (positive or negative) presenting an adjusted P-values higher than 0.05”.

The number of autocorrelations has been assessed to inform about the number of occurrences of original (i.e., non autocorrelated) features compared to the others. The mean number of such occurrence of original results are presented at the feature and at the patient levels in the Supplementary Table 3 (Part A and B, respectively). The details of the occurrence of original features (not correlated) are given in Part C and synthetised in Part D.

The mean value of observed originality among patients (no correlation, low redundancy) is 5.68 when considered every intersection together. The highest originality is reached by the comparison between the Spatial Variability of the Alpha band and: i) the Dominant Frequency within the Alpha band or the Determinism (Part C).

At the feature-level, we observed that the existence of group of features (spectral analysis, short-term complexity) induced a strong autocorrelation and a relative lack of originality, notably for Absolute value and even more for Relative power ratio. The spatial variance group is less impaired, suggesting that these features are more original compared to the others. The most original feature is the dominant frequency in the Alpha band which has indeed been built independently from the other markers and is therefore more independent. However, this feature was poorly involved in the results of Analysis N°1 and N°2, what should apply that being original might not be sufficient to be clinically relevant.

At the subject-level, no clear pattern appears: the number of favourable outcome (Part B) is well-balanced between a high number of original values (2/4, in red) and a low number of original values (4/10, in blue).

Altogether, it appeared that the highest number of original data could be obtained by the comparison between spatial variability and the three other groups (spectrum, then short-term complexity, then power).

This result is in line with the Analysis N°1. It corroborates in particular the validity of the choice provided by the data-driven analysis in which the models for the 5 factors have selected EEG parameters related to:

- spatial variability and spectrum in 3 cases ("Behavioural circadian rhythmicity", "Hormonal circadian rhythmicity" and "Functional outcome").
- spatial variability and complexity in 1 case ("Disorders of consciousness").
- spatial variability, spectrum and power in 1 case ("Behavioural cortical Function")

They are also in accordance with the Analysis N°2 as the 4 EEG features whose circadian (24h) rhythmicity was regarded as normal (among healthy subjects: Alpha and Beta Spatial Variability, Determinism and DFA) were also selected among the most original comparison between EEG features:

- at the group-level (spatial variability and complexity, see above)
- at the feature-level (Spatial Variability of the Alpha band and Determinism, see above).

Supplementary data 3: EEG correlates of the "Disorders Of Consciousness" by the comparison between healthy participants and DOC features

The existence of **"Disorders Of Consciousness"** at the assessment (i.e. 21 DOC patients with no response to simple command vs 3 healthy participants, see Supplementary Figure 6; Table 1-C) was associated with 3 EEG parameters (*AIC* = 8; p = 0.00023; *FPR* = 0.0275; *LR* = 35.34). These 3

parameters (detailed in Supplementary Figure 6) indicated that *the 24h long-term rhythmicity of EEG* was more likely ultradian than circadian (concerning signal complexity) and DOC had *a lower fluctuation magnitude* (concerning spatial variability).

Supplementary data 4: Daily rhythms description for environmental recordings

At least one environmental parameter had a 24-hour rhythm for every patient and healthy participant, indicating that a synchronisation to environment was possible even in the ICU setting (light: 24/24 recordings; sound and light: 18/24 recordings, see Supplementary Table 4). As this assessment was discriminant neither across the groups of DOC patients and healthy participants nor within the DOC group, the correlations between behavioural and environmental assessments were scrutinised. At least one correlation (corrected p-values) between eye-opening moments and an environmental assessment (among sonometer, luxmeter, and nursing) was found for the 4 patients presenting a “homogeneous presence of all circadian rhythms” pattern.

Supplementary data 5: Phase relationship between circadian rhythmicity

The phase angle between acrophase was calculated for 7 patients presenting a hormonal circadian rhythm and another circadian rhythm.

In the group of patients with a “homogeneous presence of all circadian rhythms”, the phase angle between melatonin and cortisol peaks was 6h for Patient 1, 7h for Patient 2, 2h for Patient 3 and 4h for Patient 4. For EEG features, the phase relation was close to the melatonin peak for 3 patients (Patient 1 and 2 when considering DFA and Determinism; Patient 4 when considering Beta Spatial Variability) and with the cortisol peak for Patient 3 only (considering Alpha and Beta Spatial Variability). The eye-closing maximum was in a close phase relation with the melatonin peak for Patient 1-2 and with the cortisol nadir for Patient 3-4.

In the group of patients with a “heterogeneity in the presence of circadian rhythms”, the melatonin and cortisol curves did not present concomitantly a reliable circadian fit allowing to calculate a consistent phase angle. The melatonin peak had a close phase relation with the eye-closing maximum for Patient 16 and with EEG (considering DFA and Alpha Spatial Variability) for Patient 18. On the opposite, Patient 17 presented no specific phase relation between EEG and hormones.

Supplementary data 6: Insights from the presence among DOC of circadian rhythms that are absent in healthy participants

Thirteen patients had an abnormal circadian EEG pattern (i.e. at least one feature was different from the ones of healthy participants, Supplementary Figure 5: red-squares, illustrated on Supplementary Figure 2), or an abnormal ultradian pattern for EEG (Supplementary Figure 5: red-squares and Supplementary Figure 3), and/or behaviour (Supplementary Figure 5: orange-squares and Supplementary Figure 2). No acute patient with at least one abnormal pattern (ultradian or circadian) was in the category “homogeneous presence of all circadian rhythms”.

Using a mechanistic approach focused on the Alpha-band modulation in Power and Dominant frequency, some abnormal circadian rhythms (i.e. different from healthy participants) were related to lesion topography (Supplementary Figure 7).

High values of circadian rhythm fits were observed for 12 features but abnormal circadian rhythms had an equivocal predictive value for most features because they were alternatively observed for patients with a favourable or an unfavourable outcome: fluctuations of the Alpha Dominant Frequency was the only one systematically associated with a favourable outcome (i.e. patients were Exit-MCS at least); fluctuations of the Alpha Relative Power was the only one systematically associated with an unfavourable outcome (unfavourable outcome as a composite of Coma, VS, and MCS).

For the two patients with a favourable outcome (Patient N°7 and Patient N°10) presenting a strong circadian fit for Alpha Dominant Frequency (but a non-circadian Alpha Relative Power), the lesions were multifocal and involved only the subcortical white matter (hypoxia), the corpus callosum

(hypoxia and traumatic brain injury), and the thalamo-mesencephalic junction (traumatic brain injury). On the contrary, for the three other patients (Patient N°8, Patient N°9, and Patient N°16) with an unfavourable outcome and a strong circadian fit for the Alpha Relative Power (but a non-circadian Alpha Dominant Frequency), the lesions were diffuse and related to a post-cardiac arrest encephalopathy with a comparable MRI pattern (anoxo-ischemic lesions in basal ganglia and cortical areas).

Supplementary data 7: Complementary predictive analysis for qualitative dichotomic parameters (Supplementary Table 5)

No predictive value of abnormal rhythms appeared visually or statistically for the prognostication of favourable outcome. The existence of "abnormal circadian EEG pattern" was only more frequently observed for patients evolving to UWS/VS but the difference was not significant (Table 1, PPV= 83,3%, Fisher's Exact test, $p = 0.107$). Notably, neither clinical nor neurophysiological (cortical EPs or ERPs) markers had a prognostic value for a favourable outcome in this setting.

The predictive value for the "awakening" outcome (i.e., if including the MCS/CMS within the favourable outcome group, Supplementary Table 5, right-part) was limited. The single parameter significantly associated with this outcome was the "mechanism of lesion" as most TBI patients could evolve to at least the outcome MCS/CMS. However, one could note that the mechanism of lesions was counterintuitively introduced at the end of the classification tree (Fig. 7) for statistical purposes because this marker had no association with functional outcome (Fisher's Exact test, $p = 0.294$) but was accurate (with PPV = 84.6% and NPV = 80%) for the "awakening" outcome (Fisher's Exact test, $p = 0.022$; FPR= 0.1187; LR= 7.43). Indeed, only 2 patients with a multifocal lesion did not evolve toward at least MCS (none of them had a TBI). When applied in the group of Coma + UWS/VS patients presenting a reactivity on EEG but without a <u>"homogeneous presence of all circadian</u>

rhythms", the existence of TBI was discriminant to predict any level of awakening (including MCS and independently from disability).

Notably, no brainstem (BAEPs), primary cortices (SEPs and MLAEPs), or secondary/associative cortices (N100, MMN, P300) neurophysiological response was significantly associated with the awakening outcome in this setting. The result was not modified if the standard criteria (any presence of neurophysiological responses) or a more stringent criteria (normal morphology of the neurophysiological response) was considered.

Supplementary data 8: Complementary predictive analysis for quantitative continuous parameters (Supplementary Table 6)

Clinical variables were poorly associated with outcome in univariate analysis using a general linear model (age: p = 0.0815; initial GCS: p = 0.492; initial pupillary state: p = 0.630; GCS at recording: p = 0.623), except for the score of the CRS-R at the date of recording (p = 0.0353, not independent from other clinical markers in multivariate analysis). No combination between two clinical markers outperformed the CRS-R at the date of recording. The combination of two clinical markers underperformed the combination between non-clinical markers or between clinical and non-clinical markers.

A single neurophysiological parameter – the *Permutation Entropy* for Beta Spatial Variability – outperformed systematically the others and appeared in every combination of two markers (in abscises for Supplementary Figure 8). Every patient with a favourable outcome had lower values of *Permutation Entropy* for this EEG feature. However, a significant overlap remained using this single dimension but three combinations of markers including this EEG parameter were significantly associated with the outcome (Supplementary Table 6, Supplementary Figure 8). The GCS at the date of admission (p = 0.0027; FPR= 0.036; LR= 26.8), the Alpha Absolute Power (p = 0.0019; FPR= 0.0304; LR= 31.86), and the fit of Cortisol (p = 0.0023; FPR= 0.0333; LR= 29.04) were respectively

the clinical, EEG, and hormonal makers whose combinations with *Permutation Entropy* for Beta Spatial Variability were the most synergic.

Supplementary note 1: Complementarity of short and long-term EEG metrics (as defined in Analysis N°1) to illustrate the difference between local and global states of consciousness

In the absence of behavioural signs of fluctuations, coma is commonly described as a steady condition. In chronic DOC, only few studies have investigated the fluctuations of these dimensions during a 24-hour time window for diagnostic purposes with conflicting results and different methods of wakefulness assessments[1, 2]. In the acute/sub-acute phase of DOC managed in the intensive care setting[3, 4], only limited evidence has been provided using prolonged EEG acquisitions to integrate the sleep-wake cycle information in the outcome evaluation. Several pitfalls should be considered in these studies, which may reduce their impact : i) the lack of a continuous time-line perspective to assess fluctuations directly (i.e. ordinal classification based on the "best" observed pattern)[3, 4]; ii) the EEG analysis is usually reduced to sleep-like patterns (no quantitative metrics were performed to ensure the reproducibility of the results)[3, 4]; iii) the inappropriate management of the circadian information issued from clock-controlled hormones which was performed at the population rather than the individual level[4]. The original dichotomy between the short and *long-term* scales of analysis might be reconciled by considering the theoretical proposition made by Bayne *et al.* [5] about the articulation between the global states and the local states of consciousness. In this theory, the global states of consciousness are associated with the generators of wakefulness modulation, as the authors stated: "If arousal is conceptualised in behavioural terms, then it might function as one dimension along which global states differ. If, however, it is conceptualised in neuroanatomical terms – for example, in terms of activity in the brainstem 'arousal system' – then it might function as a common cause of variation in multiple dimensions of consciousness and in so doing provide a partial explanation of certain aspects of global states of consciousness". Thus, assessing these continuous changes of global state (as "an organism can be in only one global state of consciousness at a time") would require prolonged recordings able to evaluate the co-variations of several local states of consciousness at the same time (referred to as contents of consciousness, i.e. multidimensional

experiences and thoughts). Therefore, changes of global state might be accurately described by the *24h-long-term metrics of fluctuations* used herein. Interestingly, the results of the present study may indicate that the *24h long-term changes of the global states of consciousness* (e.g. for Determinism and Alpha Relative Power in Fig. 3-B) could be favourable when predictable and less complex because they tend toward ultradian or circadian rhythmicities. The opposite role of short-term complexity in the scientific literature (that is favourable when high with a low predictability) might be related to the complementary dimension of local states of consciousness: to have a rich content of consciousness, a complex, highly entropic, and therefore unpredictable brain function would be required at the opposite temporal scale. Altogether, the re-emerging routine of a *predictable 24h long-term rhythmicity* might be a long-range physiological prerequisite for the recovery of global states of consciousness able to promote the highest cognitive levels within each local state of consciousness.

Supplementary note 2: Implications of Analysis N°2 for the consciousness embodiment hypothesis

F. Varela has introduced the "embodiment of mind" hypothesis[6] in cognitive neuroscience of consciousness. Instead of regarding the relationship between consciousness and its correlate as a "one-way causal explanation", he proposed a "two-way account" in which consciousness "emerged from the organisation of complex systems" in interaction with the environment. Due to this genealogy, the conscious process transcended the divisions between brain, body, and world as it was partly determined by each of them. From a pragmatic point of view, this theory defines the "readiness for action" from the enaction principle. The neural conscious processes require a high responsiveness to the environment to remain viable over prolonged period. For example, in ecological conditions, integrating inputs from the outside is fundamental to face immediate situations of danger. Considering this integrated physiological issue, enaction explains how both alertness and cognitive ability could be immediately unified[7]. The favourable outcome observed in patients presenting "homogeneous circadian rhythms" could reflect that a resilient harmonious relationship between the brain and body

is a prerequisite to consciousness recovery (body-brain-environment interactions being first expressed by reflexive behaviours and then by adapted ones). How these results could be translated into a more comprehensive assessment of prognosis in the intensive care setting remains to be defined logistically but the present results emphasise that this direction of research would be complementary to tracking signs of cortically-mediated behaviour[8] related to a favourable outcome[9], with a possible synergic predictive value.

Supplementary note 3: Theoretical relationship between predictability and rhythmicity in Analysis N°1 and Analysis N°2

In our work, the relationship between predictability and rhythmicity was found incidentally but was not hypothesised a priori. It is not trivial to explain: to have both a high ultradian fit and a high predictability, the *24h long-term fluctuation pattern* has to be regular and sinusoidal enough to correlate with the expected theoretical sinusoidal shape at the considered period, which is specific to each patient. For this reason, the circadian rhythm (which represents the basis of Analysis N°2 but for which a single variable is studied in Analysis N°1) cannot be qualified as predictable at the 24h scale since no recurrence is allowed at this measurement scale for this fluctuation period. To evaluate whether circadian rhythms are predictable, the measurement scale would need to be of several days or weeks. More theoretically, predictability and rhythmicity can be regarded as related to the two complementary ways of describing EEG complexity proposed by Lau et al.: rhythmicity stands as a particular sinusoidal manner of assessing regularity (defined as "the general amount of repetitions of patterns in the system's trajectory") while predictability is defined as a non-linear "temporal evolution of the system states"[10].

Supplementary note 4: Usefulness of cerebral and eye-opening/closing fluctuations during coma

Fluctuations have been considered so far from a sleep-like or circadian-like perspective, but fluctuation features *per se* have rarely been described. Therefore, comparative analyses of wakefulness and awareness fluctuations have not been performed.

24h long-term fluctuations have been historically described in post-coma patients using neurophysiological changes of muscular tonus or EEG rhythms[11, 12] and some EEG fluctuations were interpretated as sleep episodes by these authors. The observation of transient sleep moments has been confirmed in the acute context with a prognostic value based on an ordinal classification[3]. However, as background EEG rhythms are modified by the DOC themselves[13], standard rules of sleep-like classification based on healthy participants have been considered ineffective to define normative data for pathological cases[14, 15, 16]. Complete hypnograms have been built for chronic patients[17] with inconsistent results between studies, even from the same group[15]. The possibility of reliably classifying sleep moments has been described by few authors even among UWS/VS patients[18] and recused by others[16], leading to the development of alternative deep-learning methods[19].

The alternation of eye-opening and eye-closing moments may denote a self-evident clue of behavioural wakefulness. As soon as the ability of spontaneous eye-opening is restored, the disappearance of this behavioural marker of wakefulness may be interpreted as sleep moments, based on the former definition of the vegetative state (i.e. before separating MCS/CMS patients from this group[20, 21]). However, this obvious equivalence between the static evaluation of wakefulness and the actual behaviour-neurophysiology relationship may be lacking in specific contexts, such as during the ontological development of consciousness in early childhood[22, 23] or in cases of pathological states of consciousness[14, 24].It was proposed for example that UWS/VS patients had in fact no real changes of neurophysiological wakefulness despite behavioural variations, contrary to MCS/CMS patients[17]. But this result was not confirmed in a larger population of 20 DOC patients[15] and many UWS/VS patients have been previously demonstrated to display modulable sleep-like patterns[1, 15, 25].

In the present study, we showed that in case of UWS/VS-like behavioural phenotype (**"behavioural circadian rhythmicity"**[20]), the EEG pattern displayed some pseudo-ultradian characteristics of spectral results that could be used to assess wakefulness changes without evaluating

behaviour directly, despite the fact that the minimal EEG duration required to do so remains unclear. These results infirmed the previous claim that UWS/VS patients could present no significant fluctuation of EEG. However, they showed a heterogeneous profile with a paradoxical *low 24h long-term predictability* for a spatial variability feature, which was in opposition to the patterns of *high 24h long-term predictability* of "favourable profiles", as if a circadian rhythmicity restricted to a non-cortically driven wakefulness behaviour had neither the same physiological background nor an equivalent clinical significance.

Supplementary note 5: Using the Flip-Flop model impairment to interpret the difficulties of patients' evaluation in clinical routine

In the present context, fluctuations with a sharp up-and-down shape could be compatible with the persistence of a weak and/or unstable system regulating the sleep-wake cycles. In other words, a reduction of wake inputs in the Flip-Flop balance may destabilise the length of each oscillation[26], leading to accelerate the metronome switching the state of wakefulness. Among DOC patients, the transitions between these states (assessed by EEG) would remain as sharp as the transitions occurring within normal sleep (in absence of peculiar homeostatic conditions leading to sleep inertia[27]) in line with the sleep-wake switch model proposing that "there are obvious adaptive advantages to a wake–sleep system that makes rapid and complete transitions, as it would be dangerous for animals to have impaired alertness while engaging in waking behaviours, and inefficient for them to spend their sleep periods half-awake"[26]. Consequently, it becomes highly unstable in DOC patients, as it perpetually tends toward the recovery of another state of wakefulness that never remains steady. Moreover, this instability is associated with a high predictability of fluctuations because the conditions of each switch are constant.

This instability of wakefulness might have led to define awareness as being "minimal" for some patients, in spite of the singularity of the conscious process[8]. We propose that the in-between state of post-coma might rather be described as "fragile" than "minimal": an instable wakefulness on

the way to recovery could negatively interact with impaired cognitive processes due to direct telencephalic lesions or to the disruption of diffuse neuromodulation projections from the brainstem monoaminergic and cholinergic structures[26]. These altered cognitive functions would be inconstantly observed because of a failed Flip-Flop system but these occurrences might be more probable when the wakefulness dynamic reaches its highest level. Then, as soon as the underlying structures involved in this Flip-Flop system become active, recovery may engage in a virtuous circle, as illustrated in a longitudinal study involving traumatic patients emerging from disorders of consciousness (corresponding to MCS plus or Exit-MCS[28]) in which cognition improvement was concomitant to the quality of the sleep-wake cycles. In accordance with the recent proposal that circadian recovery may be a prerequisite to cognitive improvement in comatose patients[2], a functional circadian timing might be required to stabilise the sleep-wake switch and to restore 24-hour behavioural cycles. These concomitant mechanisms could explain a robust cognitive recovery indicated by goal-directed behaviours.

Supplementary note 6: Interpretation per feature: Spectral analysis

The fluctuations were observed with both Absolute and Relative Power features. The variations of low frequency (Delta- and Theta-bands) were different from those of high frequency (Alpha- and Beta-bands). It explained the sensitivity of the AR4 model to overall changes of EEG spectrum. DOC patients had reduced variations of low frequency in general, presumably due to a lower best EEG background rhythm[29]. Among the patients presenting a behavioural circadian rhythm, the amplitude of the spectral fluctuations was high with a short period in the Delta band (Fig. 4-A) while a high amplitude of Alpha Absolute Power variation was correlated to the outcome (Fig. 3-B).

The role of Alpha Power for diagnosis (e.g. the low *Permutation Entropy* for Alpha Relative Power indicating a high *long-term predictability* in the MCS/CMS group) or prognosis (e.g. the high *Standard deviation* for Alpha Absolute Power observed in the favourable outcome group) has been

reported previously using either the static power ratio[30, 31], the day-night contrast[16], or the richness of the spatial repertoire in the Alpha-band connectivity[32]. The preeminent place of Alpha Relative Power in the ability to classify DOC has been recently emphasised in a multimodal and multicentre study[33]. Despite the strength of the Alpha-band analysis alone, complementary assessments of connectivity or complexity allowed more robust classifications, which were valuable to be extrapolated to other centres.

Conversely, the observations made among the patients presenting a **"behavioural circadian rhythm"** emphasised that ultradian oscillations of EEG could concern the spectral analysis and not only the spectral entropy[30]. The ultradian fluctuations in the Beta band were particularly associated with the **"hormonal circadian rhythmicity"**. Despite its interest, this result cannot be compared *per se* to published studies. Indeed, even the most recent and specific studies about circadian rhythms during coma have not compared the EEG pattern to the hormonal markers of SCN function. Most studies have focused on defining the associations of clinical groups using temperature[34], actigraphy[35], day/night changes of EEG[16], or a combination of factors (such as actigraphy, temperature, and melatonin[36]). Isolated hormonal results have been reported in the acute[37] and chronic[38] settings. Longitudinal studies have implicated hormonal[2] and EEG[28] assessments independently to show that the cognitive recovery entailed a circadian rhythmicity. However, no study so far has addressed a comparable neurophysiological association of hormonal dosage with the explicit aim to extrapolate the actual neurobiological function of the SCN master clock.

It should be noticed that the period of neurophysiological fluctuations was shorter in presence of a **"hormonal circadian rhythm",** as if EEG rhythms were less resilient to circadian disruptions and presented in the first place a circadian decay leading to a BRAC-like (Basic Rest-Activity Cycle) ultradian oscillation[39].

Supplementary note 7: Interpretation per features: Spatial analysis

Most Spatial Variability features presented a similar pattern: the *24h long-term predictability* of the Delta, Alpha, and Beta Spatial Variability was associated with physiological or favourable

patterns. Importantly, the profile of Spatial Variability in high frequency bands (Alpha and Beta) were key for several results. First, their circadian fit defined the "normality" for the neurophysiological circadian rhythms (related to the outcome in case of "homogeneous presence of all circadian rhythms"). Second, they had a predictive value of favourable **"functional outcome"** (*high fluctuation magnitude* of Alpha Spatial Variability and *low 24h long-term complexity* of Beta Spatial Variability). An inverse relationship was observed only for the Theta Spatial Variability because its *24h long-term complexity* (and not predictability) was higher for patients presenting a **"behavioural circadian rhythm"**.

No such *24h long-term* Spatial Variability metric does exist in the literature for prolonged coma assessment to allow proper comparisons. Nonetheless, indirect analogy may be done for some connectivity metrics[32, 40] that took into account the topographical issues in a more integrated method or for simpler measures of heterogeneity[16]. A previous study has reported an opposite pattern for Alpha and Theta bands with a reduced and increased local network efficiency, respectively[32]. This effect involved the clustering coefficient, which was higher in the Theta-band for DOC patients while healthy participants had a higher network efficiency in the Alpha-band. The Theta-band pattern was opposite to the Alpha-band but similar to the Delta-band when considering topological (normalised mutual information between brain networks) and topographical (modular span) variability in the spatial domain.

For Sitt *et al.*, even if *long-term fluctuation magnitude* could not strictly stand for *long-term complexity,* the metric assessing the Theta connectivity (weighted symbolic mutual information) fluctuated with a higher magnitude (standard deviation) in the MCS/CMS group[40]. Of note, in the present study, the *Standard Deviation* for Theta Spatial Variability was not different depending on the **"behavioural circadian rhythmicity"** (not shown). Therefore, the observation of a paradoxically complex Theta Spatial Variability appeared as specific to the *Permutation Entropy* variable.

The use of a ratio of asymmetry for day-night EEG patterns in chronic DOC patients may also be regarded as a kind of spatial variability assessment[16]. Slow waves were clearly different between day (high asymmetry) and night (low asymmetry) for healthy participants. This day-night difference

was lower or absent for DOC patients. It implied that normal slow waves had a higher spatial homogeneity during sleep (and consequently a lower Spatial Variability in the Delta-band) and that most moments with reduced Spatial Variability could be regarded as potential sleep phases. In the same vein, the clues indicating the absence of consciousness in the present study consisted in a lower fluctuation magnitude for the Theta and Delta Spatial Variability. One could argue that each down phase of variability metrics may correspond to moments of slow waves “sleep-like” patterns. Thus, each switch from low to high Spatial Variability could be associated with a change of stage within the repertoire of impaired wakefulness EEG rhythms[16]. Future studies should be specifically dedicated to the visual inspection and systematic comparison of the dynamic of the EEG features during these singular instants of wakefulness swings.

Supplementary References

1. Rossi Sebastiano D, *et al.* Sleep patterns associated with the severity of impairment in a large cohort of patients with chronic disorders of consciousness. *Clin Neurophysiol* **129**, 687-693 (2018).

2. Gobert F, *et al.* Is circadian rhythmicity a prerequisite to coma recovery? Circadian recovery concomitant to cognitive improvement in two comatose patients. *J Pineal Res* **66**, e12555 (2019).

3. Valente M, *et al.* Sleep organization pattern as a prognostic marker at the subacute stage of post-traumatic coma. *Clinical neurophysiology : official journal of the International Federation of Clinical Neurophysiology* **113**, 1798-1805 (2002).

4. Yang X-a, *et al.* Prognostic roles of sleep electroencephalography pattern and circadian rhythm biomarkers in the recovery of consciousness in patients with coma: a prospective cohort study. *Sleep Medicine*, (2020).

5. Bayne T, Hohwy J, Owen AM. Are There Levels of Consciousness? *Trends Cogn Sci* **20**, 405-413 (2016).

6. Thompson E, Varela FJ. Radical embodiment: neural dynamics and consciousness. *Trends Cogn Sci* **5**, 418-425 (2001).

7. Ceruti M, Damiano L. Plural Embodiment(s) of Mind. Genealogy and Guidelines for a Radically Embodied Approach to Mind and Consciousness. *Front Psychol* **9**, (2018).

8. Naccache L. Minimally conscious state or cortically mediated state? *Brain*, (2017).

9. Faugeras F, *et al.* Survival and consciousness recovery are better in the minimally conscious state than in the vegetative state. *Brain Inj* **32**, 72-77 (2018).

10. Lau ZJ, Pham T, Chen SHA, Makowski D. Brain entropy, fractal dimensions and predictability: A review of complexity measures for EEG in healthy and neuropsychiatric populations. *Eur J Neurosci* **56**, 5047-5069 (2022).

11. Jouvet M, Pellin B, Mounier D. [Polygraphic study of the different phases of sleep during chronic consciousness disorders (prolonged comas)]. *Revue neurologique* **105**, 181-186 (1961).

12. Chatrian GE, White LE, Jr., Daly D. Electroencephalographic patterns resembling those of sleep in certain comatose states after injuries to the head. *Electroencephalography and clinical neurophysiology* **15**, 272-280 (1963).

13. Schiff ND, Nauvel T, Victor JD. Large-scale brain dynamics in disorders of consciousness. *Curr Opin Neurobiol* **25**, 7-14 (2014).

14. Cologan V, Schabus M, Ledoux D, Moonen G, Maquet P, Laureys S. Sleep in disorders of consciousness. *Sleep Medicine Reviews* **14**, 97-105 (2010).

15. Cologan V, *et al.* Sleep in the Unresponsive Wakefulness Syndrome and Minimally Conscious State. *Journal of neurotrauma*, (2013).

16. Wislowska M, *et al.* Night and day variations of sleep in patients with disorders of consciousness. *Scientific reports* **7**, 266 (2017).

17. Landsness E, *et al.* Electrophysiological correlates of behavioural changes in vigilance in vegetative state and minimally conscious state. *Brain : a journal of neurology* **134**, 2222-2232 (2011).

18. Pavlov YG, *et al.* Night sleep in patients with vegetative state. *J Sleep Res* **26**, 629-640 (2017).

19. Wielek T, *et al.* Sleep in patients with disorders of consciousness characterized by means of machine learning. *PLoS One* **13**, e0190458 (2018).

20. PVS TM-STFo. Medical aspects of the persistent vegetative state (1). *The New England journal of medicine* **330**, 1499-1508 (1994).

21. Giacino JT, *et al.* The minimally conscious state: definition and diagnostic criteria. *Neurology* **58**, 349-353 (2002).

22. Curzi-Dascalova L. [Waking and sleeping E.E.G. in normal babies before 6 months of age (author's transl)]. *Rev Electroencephalogr Neurophysiol Clin* **7**, 316-326 (1977).

23. Lynch JA, Aserinsky E. Developmental changes of oculomotor characteristics in infants when awake and in the 'active state of sleep'. *Behav Brain Res* **20**, 175-183 (1986).

24. Bekinschtein T, Cologan V, Dahmen B, Golombek D. You are only coming through in waves: wakefulness variability and assessment in patients with impaired consciousness. **177**, 171-189 (2009).

25. Bedini G, *et al.* Is Period3 Genotype Associated With Sleep and Recovery in Patients With Disorders of Consciousness? *Neurorehabil Neural Repair* **30**, 461-469 (2016).

26. Saper CB, Scammell TE, Lu J. Hypothalamic regulation of sleep and circadian rhythms. *Nature* **437**, 1257-1263 (2005).

27. Hilditch CJ, McHill AW. Sleep inertia: current insights. *Nat Sci Sleep* **11**, 155-165 (2019).

28. Duclos C, *et al.* Parallel recovery of consciousness and sleep in acute traumatic brain injury. *Neurology* **88**, 268-275 (2017).

29. Lechinger J, *et al.* CRS-R score in disorders of consciousness is strongly related to spectral EEG at rest. *J Neurol* **260**, 2348-2356 (2013).

30. Piarulli A, Bergamasco M, Thibaut A, Cologan V, Gosseries O, Laureys S. EEG ultradian rhythmicity differences in disorders of consciousness during wakefulness. *J Neurol* **263**, 1746-1760 (2016).

31. Lehembre R, *et al.* Resting-state EEG study of comatose patients: a connectivity and frequency analysis to find differences between vegetative and minimally conscious states. *Funct Neurol* **27**, 41-47 (2012).

32. Chennu S, *et al.* Spectral signatures of reorganised brain networks in disorders of consciousness. *PLoS Comput Biol* **10**, e1003887 (2014).

33. Engemann DA, *et al.* Robust EEG-based cross-site and cross-protocol classification of states of consciousness. *Brain* **141**, 3179-3192 (2018).

34. Blume C, *et al.* Significance of circadian rhythms in severely brain-injured patients: A clue to consciousness? *Neurology* **88**, 1933-1941 (2017).

35. Cruse D, *et al.* Actigraphy assessments of circadian sleep-wake cycles in the Vegetative and Minimally Conscious States. *BMC Med* **11**, 18 (2013).

36. Blume C, *et al.* Healthier rhythm, healthier brain? Integrity of circadian melatonin and temperature rhythms relates to the clinical state of brain-injured patients. *Eur J Neurol* **26**, 1051-1059 (2019).

37. Paparrigopoulos T, *et al.* Melatonin secretion after head injury: a pilot study. *Brain Inj* **20**, 873-878 (2006).

38. Guaraldi P, *et al.* Nocturnal melatonin regulation in post-traumatic vegetative state: a possible role for melatonin supplementation? *Chronobiol Int* **31**, 741-745 (2014).

39. Kleitman N. Basic rest-activity cycle--22 years later. *Sleep* **5**, 311-317 (1982).

40. Sitt JD, *et al.* Large scale screening of neural signatures of consciousness in patients in a vegetative or minimally conscious state. *Brain* **137**, 2258-2270 (2014).
