# Supplementary material for: Twenty-four-hour rhythmicities in disorders of consciousness are associated with a favourable outcome
Source: Commun Biol. 2023 Nov 29;6:1213. doi: 10.1038/s42003-023-05588-2 (PMC10687012; doi:10.1038/s42003-023-05588-2)
Supplement: Supplementary file 3 — Description of Additional Supplementary Files [file 42003_2023_5588_MOESM3_ESM.docx]

Description of Additional Supplementary Files

**File name:** Supplementary Information

**Description: Supplementary Figures, Supplementary Tables, and Supplementary Text**

Supplementary Figures

- Supplementary Figure 1: *Illustration of the four rhythmic patterns existing for healthy participants and patients during coma and post-coma*
- Supplementary Figure 2: *Illustration N°2 with Patient N°14 in Figure 5*
- Supplementary Figure 3: *Illustration N°3 with Patient N°12 (first recording) in Figure 5*
- Supplementary Figure 4: *Illustration of the normal rhythms assessed in a healthy participant*
- Supplementary Figure 5: *Synthetic overview of homogeneity and heterogeneity between all circadian rhythms with details about abnormal circadian rhythms.*
- Supplementary Figure 6: *Supervised data-driven analysis concerning the parameters related to the existence of a “Disorders Of Consciousness”*
- Supplementary Figure 7: *Illustration of two EEG abnormal circadian rhythm patterns associated with an opposite outcome*
- Supplementary Figure 8: *Comparison of the best discriminative performances of quantitative clinical and neurophysiological variables for favourable outcome*

Supplementary Tables

- Supplementary Table 1: Details of descriptive statistics
- Supplementary Table 2: Details of the population description for radiological features concerning cortical lesions (A) and extra-cortical lesions (B) with a synthetic view (C) for lateralised or bilateral lesions at the system level
- Supplementary Table 3: Autocorrelations and originality between EEG features
- Supplementary Table 4: Environmental assessments
- Supplementary Table 5: Comparison of the predictive values for clinical and neurophysiological qualitative markers (dichotomic)
- Supplementary Table 6: Comparison between clinical and multimodal quantitative markers

Supplementary Text

- Supplementary data 1: Radiological features of patients’ lesions
- Supplementary data 2: Autocorrelations between EEG times series to describe the originality of features
- Supplementary data 3: EEG correlates of the “Disorders Of Consciousness” by the comparison between healthy participants and DOC features
- Supplementary data 4: Daily rhythms description for environmental recordings
- Supplementary data 5: Phase relationship between circadian rhythmicity
- Supplementary data 6: Insights from the presence among DOC of circadian rhythms that are absent in healthy participants
- Supplementary data 7: Complementary predictive analysis for qualitative dichotomic parameters (Supplementary Table 3)
- Supplementary data 8: Complementary predictive analysis for quantitative continuous parameters (Supplementary Table 4)
- Supplementary note 1: Complementarity of short and long-term EEG metrics (as defined in Analysis N°1) to illustrate the difference between local and global states of consciousness
- Supplementary note 2: Implications of Analysis N°2 for the consciousness embodiment hypothesis
- Supplementary note 3: Theoretical relationship between predictability and rhythmicity in Analysis N°1 and Analysis N°2
- Supplementary note 4: Usefulness of cerebral and eye-opening/closing fluctuations during coma
- Supplementary note 5: Using the Flip-Flop model impairment to interpret the difficulties of patients’ evaluation in clinical routine
- Supplementary note 6: Interpretation per feature: Spectral analysis
- Supplementary note 7: Interpretation per features: Spatial analysis

Supplementary References

**File name:** Supplementary File 1

**Description:** Tables of numerical values for boxplots in Figures 3, 4, 5 and Supplementary Figure 6.

**File name:** Supplementary File 2

**Description:** Tables of numerical values for times series in Figure 7
